# Regional Variability in the Care and Outcomes of Subarachnoid Hemorrhage Patients in the United States

**DOI:** 10.3389/fneur.2022.908609

**Published:** 2022-06-16

**Authors:** Vishank A. Shah, Syed Omar Kazmi, Rahul Damani, Alyssa Hartsell Harris, Samuel F. Hohmann, Eusebia Calvillo, Jose I. Suarez

**Affiliations:** ^1^Division of Neurosciences Critical Care, Department of Neurology, Anesthesiology and Critical Care Medicine, Johns Hopkins University School of Medicine, Baltimore, MD, United States; ^2^Salem Health Hospital, Salem, OR, United States; ^3^Department of Neurology, Baylor College of Medicine, Houston, TX, United States; ^4^Center for Advanced Analytics and Informatics, Vizient, Inc., Chicago, IL, United States

**Keywords:** clinical practice variation, critical care, clinical practice variability, discharge outcomes, hospital care, subarachnoid hemorrhage (SAH)

## Abstract

**Background and Objectives:**

Regional variability in subarachnoid hemorrhage (SAH) care is reported in physician surveys. We aimed to describe variability in SAH care using patient-level data and identify factors impacting hospital outcomes and regional variability in outcomes.

**Methods:**

A retrospective multi-center cross-sectional cohort study of consecutive non-traumatic SAH patients in the Vizient Clinical Data Base, between January 1st, 2009 and December 30th, 2018 was performed. Participating hospitals were divided into US regions: Northeast, Midwest, South, West. Regional demographics, co-morbidities, severity-of-illness, complications, interventions and discharge outcomes were compared. Multivariable logistic regression was performed to identify factors independently associated with primary outcomes: hospital mortality and poor discharge outcome. Poor discharge outcome was defined by the Nationwide Inpatient Sample-SAH Outcome Measure, an externally-validated outcome measure combining death, discharge disposition, tracheostomy and/or gastrostomy. Regional variability in the associations between care and outcomes were assessed by introducing an interaction term for US region into the models.

**Results:**

Of 109,034 patients included, 24.3% were from Northeast, 24.9% Midwest, 34.9% South, 15.9% West. Mean (SD) age was 58.6 (15.6) years and 64,245 (58.9%) were female. In-hospital mortality occurred in 21,991 (20.2%) and 44,159 (40.5%) had poor discharge outcome. There was significant variability in severity-of-illness, co-morbidities, complications and interventions across US regions. Notable findings were higher prevalence of surgical clipping (18.8 vs. 11.6%), delayed cerebral ischemia (4.3 vs. 3.1%), seizures (16.5 vs. 14.8%), infections (18 vs. 14.7%), length of stay (mean [SD] days; 15.7 [19.2] vs. 14.1 [16.7]) and health-care direct costs (mean [SD] USD; 80,379 [98,999]. vs. 58,264 [74,430]) in the West when compared to other regions (all *p* < 0.0001). Variability in care was also associated with modest variability in hospital mortality and discharge outcome. Aneurysm repair, nimodipine use, later admission-year, endovascular rescue therapies reduced the odds for poor outcome. Age, severity-of-illness, co-morbidities, hospital complications, and vasopressor use increased those odds (c-statistic; mortality: 0.77; discharge outcome: 0.81). Regional interaction effect was significant for admission severity-of-illness, aneurysm-repair and nimodipine-use.

**Discussion:**

Multiple hospital-care factors impact SAH outcomes and significant variability in hospital-care and modest variability in discharge-outcomes exists across the US. Variability in SAH-severity, nimodipine-use and aneurysm-repair may drive variability in outcomes.

## Introduction

Subarachnoid hemorrhage (SAH) patients have high prehospital mortality and those surviving the early phase, experience multiple hospital complications, particularly early brain injury, rebleeding, delayed cerebral ischemic (DCI), cardio-pulmonary complications, nosocomial infections, fluid imbalance and other iatrogenic complications, all of which further impact survival and functional outcomes after SAH and may be related to hospital care (1–5). SAH patients, thus require care by a multidisciplinary group of practitioners, preferably in specialized neurointensive care units (2, 3). However, despite advances in intensive care unit (ICU) care and nearly a 50% reduction in SAH mortality over the last two decades (6), limited scientific data guide therapy in SAH and very few interventions have strong evidence for impacting survival and outcomes (3). As a consequence, significant variability in SAH care is expected and has been reported in prior studies (7–12).

Majority of the studies that assess clinical practice variability in SAH are national and international practice pattern surveys (7–12), that are often subject to recall biases and do not include individual patient data and thus have not been able to determine if variability in care practices are associated with patient outcomes. We, therefore, used the Vizient Clinical Data Base (CDB), that includes patient-level data for this study. We hypothesized that there is significant regional variability in the care of SAH patients in the United States (US) and that this variability is associated with discharge outcomes. The objective of this study was to compare SAH care across different US geographic regions. We also aimed to identify hospital-care factors associated with discharge outcomes in SAH and to determine if regional variability in hospital-care is associated with outcome variability.

## Materials and Methods

### Standard Protocol Approvals, Registrations, and Patient Consents

The Institutional Review Board (IRB) of the Johns Hopkins University School of Medicine deemed the study exempt (Protocol Number: IRB00294595), given that this was a retrospective analysis of deidentified patient data. The study was performed in accordance with the Strengthening The Reporting of OBservational studies in Epidemiology (STROBE) (13) and REporting of studies Conducted using Observational Routinely-collected Data (RECORD) guidelines (14). STROBE and RECORD checklists are included in the Supplementary Material.

### Study Design and Data Source

We conducted a retrospective analysis of a cross-sectional cohort generated from the Vizient CDB. The Vizient CDB is a multi-center healthcare analytics platform for performance improvement (15) and comprises data from >95% of the US academic medical centers and their affiliated hospitals. It is a collection of patient-level Uniform Bill-04 billing data from all participating hospitals. The discharge abstract data contain information regarding patient demographics, hospital medications, procedures, hospital morbidity, discharge disposition and mortality. Vizient CDB has been previously validated in several observational studies (16–22).

### Study Population

In this study, we included all adult patients (age >18 years) with a primary diagnosis of non-traumatic SAH (ICD-9 code: 430 and/or ICD-10 code: I60) in the Vizient CDB between January 1st, 2009 and December 30th, 2018. We chose to assess variability in hospital-care across all forms of non-traumatic SAH and included non-aneurysmal SAH patients as well, in order to ensure that patients are not excluded due to misclassification, given that the ICD codes do not differentiate between aneurysmal and non-aneurysmal hemorrhages. Patients were divided into four groups, based on the US Census Bureau geographic region of the hospital they were admitted to: Northeast (NE), South (S), Midwest (MW) and West (W) (23).

### Variable Measurements

Demographics (patient age, race/ethnicity, gender), severity of illness upon admission, year-of-admission, co-morbidities, hospital complications, procedures, neuroimaging modalities, pertinent medications, hospital mortality, discharge disposition, length of stay, health-care direct costs and US census region were extracted from Vizient CDB for all patients. Categories of race and ethnicity followed the CMS methodology (24). Specific diagnoses, imaging modalities and procedures were identified using International Classification of Diseases, Ninth and Tenth Edition (ICD-9 and ICD-10, Clinical Modification [CM] codes provided in the Supplementary Material). Imaging modalities, procedures, medications were flagged as present or absent during the encounter, regardless of the number of times they were used. DCI was defined by linking ICD codes for non-traumatic SAH and cerebral vasospasm, stroke and stroke-related sequalae (Supplementary Material). Annual SAH case volume by hospital was calculated, and a flag was created for low-volume SAH centers (<35 cases annually) in each US region (3). The designation of severity of illness (SOI) upon admission was derived from a combination of the 3M All Payer Refined-Diagnosis Related Group (APR-DRG) grouper (25). The SOI and associated risk of mortality is disease-specific and uses a classification for risk stratification consisting of four severity categories: minor, moderate, major, and extreme. In SAH, the APR-DRG risk-of-mortality severity index has been demonstrated to be valid and reliable severity adjustment score (26), with good predictive accuracy (AUC: 0.75) for poor outcome after SAH (27). It has been used previously as an adjustment for severity of illness in the absence of World Federation of Neurological Sciences (WFNS), Glasgow Coma (GCS) or Hunt-Hess scales in prior studies (28, 29).

### Outcomes

Primary outcome measures included in-hospital mortality and the Nationwide Inpatient Sample-SAH Outcome Measure (NIS-SOM). NIS-SOM is a dichotomous functional outcome measure that defines good outcome as discharge to home or rehabilitation facility and poor outcome as a composite of in-hospital mortality, discharge to nursing facility/extended care facility/long-term acute care or hospice, placement of tracheostomy and/or gastrostomy (27). The NIS-SOM has been externally validated in a cohort of 716 SAH patients, where a strong correlation was noted between poor outcome defined by NIS-SOM and modified Rankin score (mRS) > 3 at discharge, with a high agreement (95%) and kappa-statistic of 0.84 (27). Secondary outcomes included hospital and ICU length of stay as well as health-care direct costs.

### Data Access and Availability Statement

Individual de-identified patient data was available to all investigators through a written agreement with the Center for Advanced Analytics and Informatics, Vizient, Inc. Access to the data can be obtained by submitting a formal proposal in writing to the Center for Advanced Analytics and Informatics, Vizient, Inc.

### Statistical Analysis

Patient demographics, comorbidities, care processes, length of stay and outcomes were stratified by US regions and summarized using frequencies (%) for categorical and means (SD) for continuous variables. Variability in these factors across regions was assessed by comparing frequencies/means using the χ^2^ test or the analysis of variance (ANOVA) tests, as appropriate. To assess the relationship between variability in care and outcomes, we first fit multivariable logistic regression models, treating the study outcomes as the response and hospital-care factors, including treatment interventions and hospital complication flag as predictors. Hospital-care factors were selected if factors were known to commonly impact SAH outcomes and/or those associated with significant variability across the US regions. In the models, we also adjusted for potential confounders including age, admission SOI, presence of any comorbidity. To adjust for temporal trends in care, we also adjusted for the year-of-admission in the models. Final covariates were chosen if a strong association was noted, judged by *p-*values (*p* < 0.05) from the Wald test. Subsequently, to assess the impact of regional variability in hospital care and outcome, an interaction effect for US region was included in the logistic regression models. This assessed the relationship between various predictors and outcome by US regions. Performance of the models was tested using a concordance (c) statistic. Statistical analyses were performed using the software SAS (version 9.4, SAS Institute, Inc., Cary, NC), and *p*-values were two-sided with <0.05 considered statistically significant.

## Results

### Baseline Characteristics

**Figure 1:**
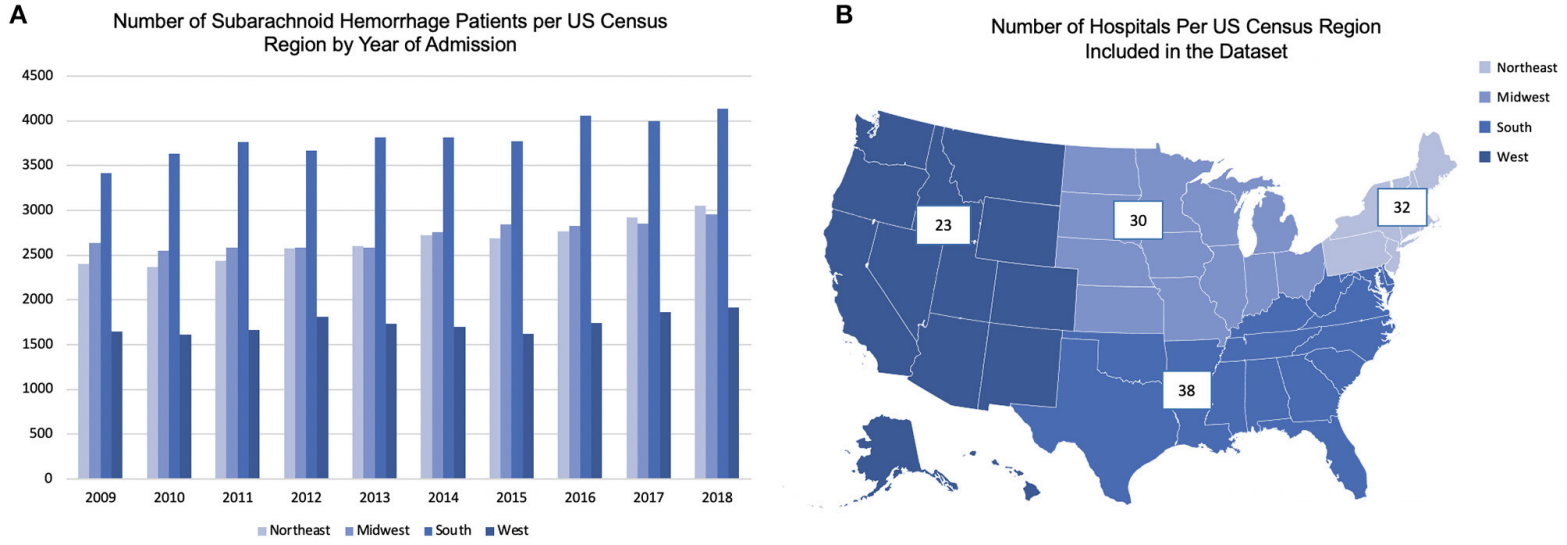
**(A)** shows distribution of patients per United States Census Bureau region per year; **(B)** shows number of hospitals included in the analysis from the Vizient CDB per United States Census Bureau regions.

We analyzed data from 109,034 non-traumatic SAH patients included in the Vizient CDB. Of these, 26,519 (24.3%) were from the NE; 27,166 (24.9%) from MW; 38,055 (34.9%) from S and 17,294 (15.9%) from W. Regional distribution of patients per year is shown in Figure 1. Number of SAH patients in the CDB increased from 2009 to 2018, which is likely due to the inclusion of more hospitals in the Vizient CDB.

Baseline characteristics by US census region are shown in Table 1. Mean (SD) age was 58.6 (15.6) years and was marginally higher in the NE (NE: 60.5 [15.9] years vs. mean age 57.9 [15.5] years in other regions). In total of 64,245 (58.9%) patients were female. In the total cohort, 68,492 (62.8%) were White, 19,786 (18.1%) were Black and 20,756 (19.0%) were other races and/or Hispanic ethnicity. Regionally, NE and MW had significantly higher proportions of White patients (NE: 66.3%; MW: 69.2%; S: 57.5%; W: 59.1%; *p* < 0.0001) whereas S had significantly higher proportions of Black patients (NE: 11.5%; MW: 17.5%; S: 28.4%; W: 6.7%; *p* < 0.0001). Higher proportions of other races/ethnicities (Asian, Pacific Islander, American Indian and Hispanic ethnicity) were noted in the W (34.2% vs. other region mean 16.5%; *p* < 0.0001).

**Table 1:** Variability in baseline characteristics and hospital care variables by US Region.

**Variables**	**NE (*n =* 26,519)**	**MW (*n =* 27,166)**	**S (*n =* 38,055)**	**W (*n =* 17,294)**	***p* value**
**Baseline characteristics and Care Variables for Subarachnoid Hemorrhage patients across United States (2009 to 2018)** ^**a**^					
Age, mean (SD), y	60.5 (15.9)	58.7 (15.6)	57.5 (15.3)	57.7 (15.7)	<0.0001
Sex – Female	15,568 (58.7)	15,990 (58.9)	22,817 (60)	9,870 (57.1)	<0.0001
Race: White	17,585 (66.3)	18,784 (69.2)	21,898 (58)	10,225 (59.1)	<0.0001
Black	3,043 (11.5)	4,766 (17.5)	10,817 (28)	1,160 (6.7)	
Other/unknown^b^	5,891 (22.2)	3,616 (13.3)	5,340 (14)	5,909 (34.2)	
No. of low volume SAH centers^c^	8 (25.0)	8 (26.7)	9 (23.7)	8 (34.8)	0.80
Patients in low volume SAH-centers^c^	547 (2.1)	1,009 (3.7)	1,713 (4.5)	1,480 (8.9)	<0.0001
Medical co-morbidities					
Hypertension	16,437 (62.0)	17,627 (64.9)	26,213 (69)	10,787 (62.4)	<0.0001
Diabetes mellitus	4,341 (16.4)	4,720 (17.4)	7,076 (18.6)	2,999 (17.3)	<0.0001
Congestive heart failure	2,235 (8.4)	2,484 (9.1)	3,057 (8.0)	1,294 (7.5)	<0.0001
Smoking	3,966 (15.0)	5,193 (19.1)	6,998 (18.4)	2,297 (13.3)	<0.0001
Admission severity-of-illness^d^					
Minor	1,047 (4.0)	1,002 (3.7)	1,384 (3.6)	513 (3.0)	<0.0001
Moderate	2,832 (10.7)	2,748 (10.1)	3,830 (10.1)	1,903 (11.0)	
Major	14,107 (53.2)	13,998 (51.5)	18,623 (49)	8,963 (51.8)	<0.0001 <0.0001
Extreme	8,144 (30.7)	8,389 (30.9)	13,250 (35)	5,570 (32.2)	
Radiology/procedures/medications^e^					
Cerebral angiogram/arteriogram	14,519 (54.8)	14,073 (51.8)	20,144 (53)	9,375 (54.2)	<0.0001
Extra-ventricular drain	5,694 (21.5)	5,927 (21.8)	9,192 (24.2)	4,181 (24.2)	<0.0001
Cerebral aneurysm clipping	2,419 (9.1)	3,500 (12.9)	4,701 (12.4)	3,246 (18.8)	<0.0001
Cerebral aneurysm coiling	2,737 (10.3)	2,668 (9.8)	4,138 (10.9)	1,603 (9.3)	<0.0001
Cerebral angioplasty	801 (3.0)	902 (3.3)	1,084 (2.9)	507 (2.9)	0.01
Ventriculo-peritoneal shunt	313 (1.2)	391 (1.4)	385 (1.0)	215 (1.2)	<0.0001
Tracheostomy	523 (2.0)	724 (2.7)	1,746 (4.6)	472 (2.7)	<0.0001
Gastrostomy tube placement	188 (0.7)	291 (1.1)	239 (0.6)	129 (0.8)	<0.0001
Electroencephalogram	3,755 (14.2)	4,005 (14.7)	4,870 (12.8)	2,771 (16.0)	<0.0001
Vasopressor and inotrope use^f^	2,964 (11.2)	3,617 (13.3)	5,306 (13.9)	2,411 (13.9)	<0.0001
Intra-arterial vasodilator therapy^g^	2,168 (8.2)	3,569 (13.1)	4,515 (11.9)	2,140 (12.4)	<0.0001
Nimodipine	2,808 (10.6)	2,904 (10.7)	4,557 (12.0)	2,015 (11.7)	<0.0001
Levetiracetam	4,192 (15.8)	4,075 (15.0)	5,587 (14.7)	2,513 (14.5)	0.0002
Phenytoin/fosphenytoin	265 (1.0)	258 (1.0)	431 (1.1)	321 (1.9)	<0.0001
Valproic acid	208 (0.8)	137 (0.5)	205 (0.5)	97 (0.6)	<0.0001
Albumin	802 (3.0)	968 (3.6)	1,575 (4.1)	693 (4.0)	<0.0001

*NE, Northeast; MW, Midwest; S, South; W, West; SD, Standard Deviation; SAH, Subarachnoid Hemorrhage; MRI, Magnetic Resonance Imaging. ^a^Unless otherwise specified, values are listed as number and percentage of total number of patients per region. ^b^Other race/ethnicity includes Asian race, Pacific Islander, Native American, Hispanic Ethnicity. ^c^Defined as annual SAH case load of less than 35 cases. ^d^Defined by the All Patients Refined Diagnosis Related Groups (APR-DRG) disease-specific risk-of-mortality index. ^e^Defined as at least one encounter for each modality or medication during hospital admission. ^f^Included intravenous norepinephrine, epinephrine, dopamine, vasopressin, dobutamine, milnirone. ^g^Included intra-arterial nicardipine, verapamil, milnirone, papaverine*.

Comorbidities are summarized in Table 1. Hypertension was the most common comorbidity and present in 71,064 (64.5%) of the total cohort, with highest proportions in the S (NE: 62%, MW: 64.9%, S: 69%, W: 62.4%, *p* < 0.0001).

In total of 91,044 (83.5%) patients were classified as either major or extreme SOI upon admission. SOI upon admission was also highly variable by region, with higher proportions of patients with major SOI in the NE (NE: 53.2%, MW: 51.5%, S: 49%, W: 51.8%; *p* < 0.0001) and extreme SOI in the S (NE: 30.7%, MW: 30.9%, S: 35%, W: 32.2%; *p* < 0.0001).

### Hospital Characteristics

Number of hospitals included from the Vizient CDB in this analysis from each US census region are shown in Figure 1. SAH data was available from a total of 123 hospitals, majority (>95%) of which were academic centers. Majority of the hospitals were classified as high-volume SAH centers (annual SAH cases > 35), with 33 (26.8%) hospitals classified as low-volume SAH centers across all regions. The W had a higher proportion of low-volume SAH centers in the Vizient CDB, but this difference was not statistically significant (Table 1). However, a significantly higher proportion of SAH patients were admitted to low-volume SAH centers in the W (NE: 2.1%; MW: 3.7%; S: 4.5%; W:8.9%; *p* < 0.0001) (Table 1).

### Medications

Comparison of medications used among SAH patients across the four US regions are shown in Table 1. We studied variability in use of nimodipine, antiepileptic drugs, vasopressors and albumin. Overall, 12,284 (11.3%) patients received nimodipine across all regions with higher use in the S and W (NE: 10.6%, MW: 10.7%, S: 12.0%, W: 11.7%; *p* < 0.0001). In total of 19,397 (17.8%) patients received antiepileptic drugs (AED), of which levetiracetam was most commonly used followed by phenytoin. With respect to regional variability, phenytoin use was twice more common in the W compared to other regions (NE: 1.1%; MW: 1.0%, S: 1.1%, W: 2.0%; *p* < 0.0001). Vasopressor and inotrope use (NE: 11.2%, MW: 13.3%, S: 13.9%, W: 13.9%; *p* < 0.0001) as well as albumin use (NE: 3%, S: 3.6%, MW: 4.1%, S: 4.0%; *p* < 0.0001) were less common in the NE compared to other regions.

### Hospital Procedures

Hospital procedures by US census regions are summarized in Table 1. Aneurysmal surgical clipping was used in 13,866 (12.7%) patients, while endovascular coiling was used in 11,146 (10.2%) of the total cohort. Surgical clipping was significantly more common in the W (NE: 9.1%; MW: 12.9%; S: 12.4%; W: 18.8%; *p* < 0.0001). In general, as shown in Table 2, higher proportions of patients with major SOI received surgical clipping and minor/moderate SOI received endovascular coiling. However, across all grades of SOI, the W had higher proportions of patients receiving surgical clipping than endovascular coiling (Table 2).

**Table 2:** Aneurysmal coiling vs. clipping by admission severity of illness (SOI) in each US region.

**Region**	**Coiling**	**Clipping**	***p*-value**
**Coiling vs. clipping by admission severity of illness (SOI)**			
**Minor SOI (n; Northeast** **=** **1,047, Midwest** **=** **1,002, South** **=** **1,384**,			
**West** **=** **513)**
Northeast	319 (30.4)	<5	
Midwest	320 (31.9)	<5	
South	455 (32.9)	<5	
West	149 (29.1)	<5	
**Moderate SOI (n; Northeast** **=** **2,832, Midwest** **=** **2,748, South** **=** **3,830**,			
**West** **=** **1,903)**
Northeast	563 (19.9)	397 (14.0)	<0.0001
Midwest	544 (19.8)	532 (19.3)	0.928
South	827 (21.6)	687 (17.9)	0.0004
West	292 (15.3)	540 (28.4)	<0.0001
**Major SOI (n; Northeast** **=** **14,107, Midwest** **=** **13,998, South** **=** **18,623**,			
**West** **=** **8,963)**
Northeast	1131 (8.0)	1397 (9.9)	<0.0001
Midwest	1136 (8.1)	2122 (15.2)	<0.0001
South	1614 (8.7)	2551 (13.7)	<0.0001
West	599 (6.7)	1782 (19.9)	<0.0001
**Extreme SOI (n; Northeast** **=** **8,144, Midwest** **=** **8,389, South** **=** **13,250**,			
**West** **=** **5,570)**
Northeast	632 (7.8)	524 (6.4)	0.004
Midwest	572 (6.8)	696 (8.3)	0.0001
South	1096 (8.3)	1258 (9.5)	0.0001
West	346 (6.2)	679 (12.2)	<0.0001

With respect to DCI rescue therapies, intra-arterial (IA) vasodilator use was noted in 12,392 (11.4%) patients of the total cohort and was more common than cerebral angioplasty (3,294 [3.0%]). Significant regional variability in intra-arterial vasodilator use was noted (NE: 8.2%; MW: 13.1%; S: 11.9%; W: 12.4%; *p* < 0.0001). Among other procedures, notably, tracheostomy was significantly more common in the S (NE: 2%, MW: 2.7%; S: 4.6%, W: 2.7%; *p* < 0.0001).

### Hospital Complications

There was significant variability in hospital complications across US regions as outlined in Table 3. Hydrocephalus (NE: 32.7%, MW: 34.2%, S: 37.9%, W: 35%; *p* < 0.0001) and cerebral edema (NE: 27.5%, MW: 26.1%, S: 35.2%, W: 29%; *p* < 0.0001) were more common in the S, whereas seizures/status epilepticus (NE: 15.2%, MW: 15.0%, S: 14.5%, W: 17%; *p* < 0.0001) and DCI (NE: 3.1%, MW: 3.8%, MW: 2.7%, W: 4.3%; *p* < 0.0001) were more common in the W.

**Table 3:** Variability in hospital complications and patient outcomes by US Region.

**Variables**	**NE** **(*n =* 26,519)**	**MW** **(*n =* 27,166)**	**S** **(*n =* 38,055)**	**W** **(*n =* 17,294)**	***P* value**
**Complications and outcomes in subarachnoid hemorrhage patients across the United States (2009-2018)** ^**a**^					
**Complications**					
Hydrocephalus	8,673 (32.7)	9,286 (34.2)	14,437 (37.9)	6,075 (35)	<0.0001
Delayed cerebral ischemia	830 (3.1)	1034 (3.8)	1024 (2.7)	737 (4.3)	<0.0001
Seizures/status epilepticus	4,025 (15.2)	4,081 (15.0)	5,515 (14.5)	2,844 (17)	<0.0001
Cerebral edema	7,304 (27.5)	7,099 (26.1)	13,382 (35.2)	5,043 (29)	<0.0001
Cerebral ventriculitis	637 (2.4)	785 (2.9)	1,084 (2.9)	655 (3.8)	<0.0001
Cardio-pulmonary complications	11,836 (44.6)	12,974 (47.8)	18,877 (49.6)	8,181 (47)	<0.0001
Neurogenic stress cardiomyopathy	408 (1.5)	446 (1.6)	539 (1.4)	304 (1.8)	0.0026
Volume/fluid overload	618 (2.3)	1,059 (3.9)	1,156 (3.0)	676 (3.9)	<0.0001
Acute myocardial infarction	3,856 (14.5)	3,968 (14.6)	4,782 (12.6)	2,279 (13)	<0.0001
Pulmonary edema	263 (1.0)	387 (1.4)	398 (1.1)	223 (1.3)	<0.0001
ARDS	213 (0.8)	262 (1.0)	462 (1.2)	356 (2.1)	<0.0001
Acute respiratory failure	8,389 (31.6)	9,272 (34.1)	14,595 (38.4)	6,010 (35)	<0.0001
Pulmonary embolism	527 (2.0)	623 (2.3)	704 (1.9)	366 (2.1)	0.0009
Deep venous thrombosis	47 (0.2)	70 (0.3)	69 (0.2)	31 (0.2)	0.0988
Hyponatremia	4,811 (18.1)	5,915 (21.8)	8,141 (21.4)	3,841 (22)	<0.0001
Acute kidney injury	3,160 (11.9)	3,587 (13.2)	4,673 (12.3)	2,418 (14)	<0.0001
Systemic infectious complications	3,908 (14.7)	4,138 (15.2)	5,483 (14.4)	3,112 (18)	<0.0001
Aspiration pneumonia	1,135 (4.3)	977 (3.6)	1,306 (3.4)	954 (5.5)	<0.0001
Sepsis	1,649 (6.2)	1,807 (6.7)	2,436 (6.4)	1373 (7.9)	<0.0001
Bacteremia	551 (2.1)	630 (2.3)	817 (2.2)	289 (1.7)	<0.0001
Clostridium difficle enteritis	607 (2.3)	646 (2.4)	692 (1.8)	450 (2.6)	<0.0001
**Discharge outcomes**					
In-Hospital mortality	5,404 (20.4)	5,304 (19.5)	7,689 (20.2)	3,594 (21)	0.0081
Home	10,724 (41.0)	10,999 (41.0)	17,334 (46.5)	7,426 (45)	<0.0001
Rehabilitation facility	5,674 (21.7)	5173 (19.3)	6278 (16.8)	2413 (15)	<0.0001
Skilled nursing facility	2,743 (10.5)	3,169 (11.8)	3,145 (8.4)	2,027 (12)	<0.0001
Long-term acute care	735 (2.8)	1,212 (4.5)	1,408 (3.8)	491 (3.0)	<0.0001
Hospice	826 (3.2)	922 (3.4)	1,326 (3.6)	380 (2.3)	<0.0001
Unknown/other	24 (0.1)	67 (0.3)	97 (0.3)	48 (0.3)	<0.0001
NIS-SOM poor outcome^b^	10,710 (40.4)	11,337 (41.7)	15,085 (39.6)	6,987 (40)	<0.0001
Length of hospital stay, mean (SD), days	14.23 (19.7)	13.60 (13.3)	14.42 (16.6)	15.66 (19.2)	<0.0001
Length of ICU stay, mean (SD), days	8.27 (9.9)	8.52 (9.7)	8.84 (10.3)	9.63 (12.6)	<0.0001
Health-care direct cost, mean (SD), US dollars	58,574 (93,223)	57,183 (59,576)	59,036 (66,179)	80,379 (98,999)	<0.0001

*NE, Northeast; MW, Midwest; S, South; W, West; ARDS, Acute Respiratory Distress Syndrome; NIS-SOM, Nationwide Inpatient Sample-Subarachnoid Outcome Measure; SD, Standard Deviation; US, United States. ^a^Unless otherwise specified, values are listed as number and percentage of total number of patients per region; ^b^defined as discharge to hospice, nursing facility, long-term acute care, death, need for tracheostomy and/or gastrostomy*.

Cardiopulmonary complications were significantly lower in the NE and higher in the S (NE: 44.6%, MW: 47.8%; S: 49.6%, W: 47.3%; *p* < 0.0001). Among these, acute respiratory failure was much more common in the S (NE: 31.6%, MW: 34.1%, S: 38.4%, W: 35%; *p* < 0.0001) and acute respiratory distress syndrome in W (NE: 0.8%, MW: 1.0%; S: 1.2% and W: 2.1%; *p* < 0.0001). Hyponatremia (NE: 18.1%, MW: 21.8%, S: 21.4%, W: 22%; *p* < 0.0001) and acute kidney injury (NE: 11.9%, MW: 13.2%, S: 12.3%, W: 14%; *p* < 0.0001) were less common in NE and more common in the W compared to other regions. Systemic infections were much more prevalent in the W (NE: 14.7%, MW: 15.2%, S: 14.4%, W: 18%; *p* < 0.0001) with a higher prevalence of aspiration pneumonia, sepsis and central nervous system infections (*p* < 0.0001).

### Outcomes

**Figure 2:**
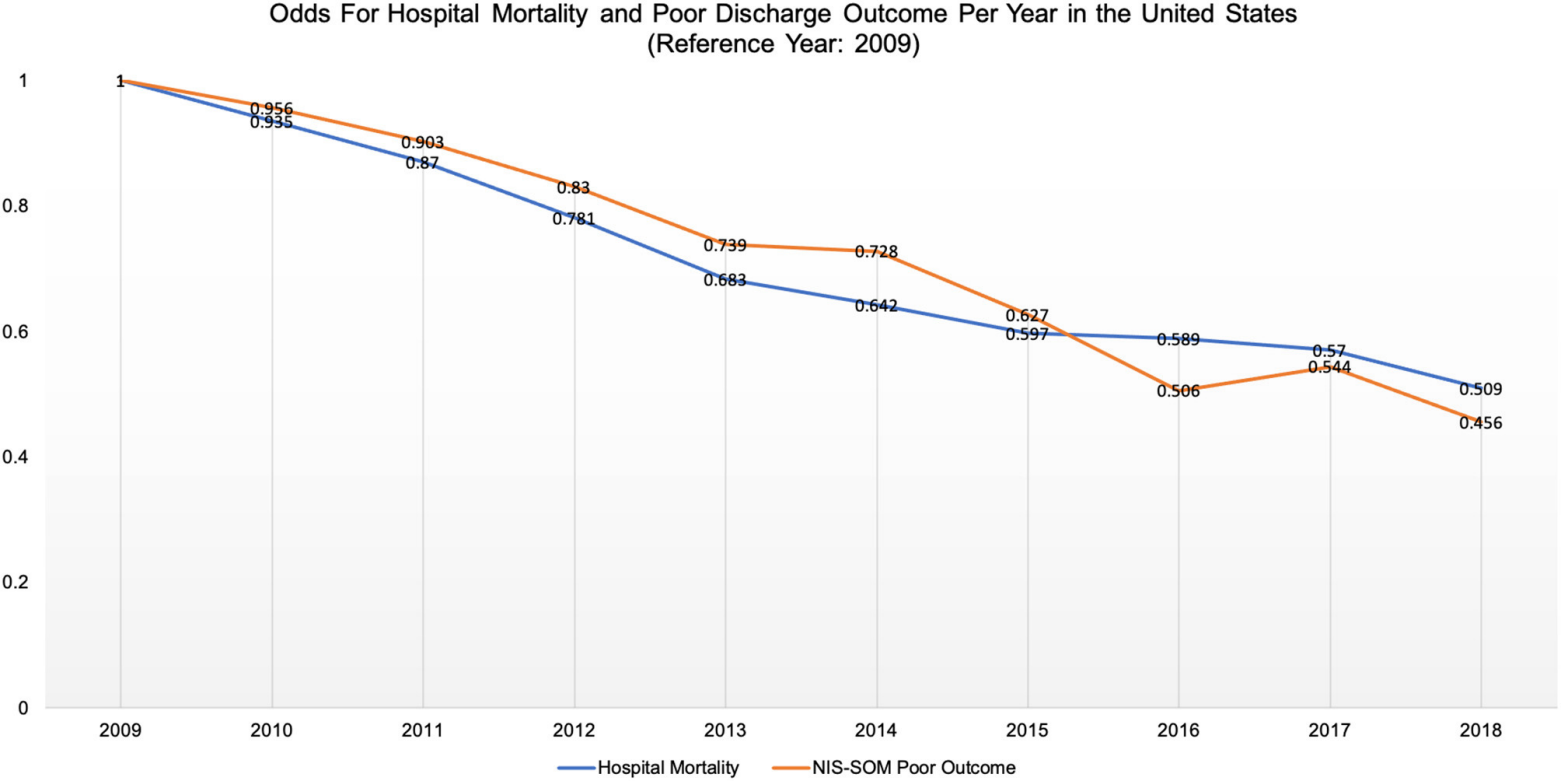
Shows sustained reduction in odds for hospital mortality and poor Nationwide Inpatient Sample SAH-Outcome Measure (NIS-SOM) per year from 2009 to 2018.

Patient outcomes are highlighted in Table 2. Overall hospital mortality occurred in 21,991 (20.2%), with modest but statistically significant variability across US regions (NE: 20.4%; MW: 19.5%; S: 20.2%; W: 20.8%; *p* = 0.004). Poor outcome by NIS-SOM occurred in 44,119 (40.5%) of the total cohort, with modest but statistically significant regional variability (NE: 40.4%; MW: 41.7%; S: 39.6%; W: 40.4%; *p* < 0.0001). Odds for hospital mortality and poor discharge outcome decreased significantly through the study period and every year in the cohort (approximately 5% per annum when compared to 2009) (Figure 2).

There was significant regional variability in discharge disposition. Overall, 43.6% patients were discharged home with highest proportions in the S followed by the W (Table 3). In total of 18.3% of patients were discharged to an acute rehabilitation facility, but significantly lower in the W: 10.4% were discharged to a skilled nursing facility with highest proportions in MW and W: 3.6% were discharged to a long-term acute care facility and 3.2% to hospice. Mean length of stay was higher in the W (mean [SD] days; NE: 14.2 [19.7], MW: 13.6 [13.3], S: 14.4 [16.6], W: 15.7 [19.2]; *p* < 0.0001), as were the ICU length of stay (mean [SD] days; NE: 8.3 [9.9], MW: 8.5 [9.7], S: 8.8 [10.3], W: 9.6 [12.6]; *p* < 0.0001) and health-care direct costs (mean [SD] USD; NE: 58,574 [93,223], MW: 57,183 [59.576], S: 59,036 [66,179], W: 80,379 [98,999]; *p* < 0.0001).

### Predictors of Hospital Outcomes

Factors independently associated with lower hospital mortality included subsequent years of admission (2014-18 vs. 2009-13: adjusted odds ratios [aOR], 0.72, 95% confidence intervals [CI], 0.69–0.74), aneurysmal repair by coiling (aOR, 0.50, 95% CI, 0.46–0.53) or clipping (aOR, 0.37, 95% CI, 0.35–0.40), presence of a co-morbidity flag (aOR, 0.75, 95% CI, 0.71–0.79), nimodipine use (OR, 0.41, 95% CI, 0.38–0.44), intra-arterial vasodilator rescue therapy (aOR, 0.48, 95% CI, 0.45–0.51) and cerebral angioplasty (aOR, 0.75, 95% CI, 0.68–0.83). Higher age (aOR per y, 1.02, 95% CI, 1.019–1.021), worse APR-DRG admission SOI (extreme vs. minor: aOR, 13.53, 95% CI, 11.60–15.78) any hospital complication flag (aOR, 1.53, 95% CI, 1.47–1.59), vasopressor use (aOR, 2.38, 95% CI, 2.24–2.54) and hospital US region West (W vs. NE: aOR, 1.12, 95% CI, 1.07–1.18) were independently associated with higher hospital mortality (c-statistic for hospital mortality model: 0.765). Regional variability in hospital mortality predictors with corresponding aOR and 95% CI by each US region are shown in Table 4. Regional interaction effect revealed that factors contributing to regional variability in hospital mortality included variability in aneurysm repair and admission SOI.

**Table 4:** Multivariable logistic regression models predicting hospital mortality by US region.

**Variable**	**Adjusted odds ratio estimates (95% confidence intervals)**				***P*** **value**	
**Hospital mortality**						
Years: 2014–2018 vs. 2009–2013	0.71 (0.69–0.74)				<0.0001	
	**Northeast**	**Midwest**	**South**	**West**	**Main effect**	**Regional interaction Effect**
Aneurysm repair						
Any procedure	0.25 (0.12–0.54)	0.24 (0.11–0.54)	0.61 (0.39–0.96)	0.68 (0.46–1.02)	<0.0001	0.001
Endovascular coiling	0.53 (0.47–0.61)	0.50 (0.44–0.57)	0.41 (0.37–0.46)	0.53 (0.45–0.63)		
Surgical clipping	0.34 (0.30–0.40)	0.33 (0.28–0.37)	0.37 (0.34–0.42)	0.42 (0.37–0.48)		
Age, per year	1.02 (1.02–1.024)	1.02 (1.02–1.021)	1.02 (1.02–1.022)	1.02 (1.02–1.023)	<0.0001	0.192
Presence of a comorbidity flag^a^	0.77 (0.69–0.85)	0.74 (0.66–0.83)	0.7 (0.66–0.80)	0.82 (0.72–0.94)	<0.0001	0.528
Severity of illness (SOI) on admission^b^						
Moderate SOI	2.36 (1.7–3.3)	1.9 (1.41–2.67)	1.91 (1.42–2.55)	1.66 (1.08–2.56)	<0.0001	0.002
Major SOI	3.25 (2.39–4.42)	2.76 (2.05–3.70)	2.92 (2.23–3.81)	2.73 (1.82–4.09)		
Extreme SOI	15.1 (11.1–20.5)	11.90 (8.9–16.0)	14.4 (11.0–18.8)	13.13 (8.8–19.7)		
Presence of a hospital complication flag^c^	1.53 (1.42–1.65)	1.64 (1.52–1.77)	1.43 (1.34–1.53)	1.58 (1.44–1.73)	<0.0001	0.068
Nimodipine use	0.40 (0.35–0.47)	0.43 (0.37–0.50)	0.39 (0.35–0.45)	0.48 (0.40–0.58)	<0.0001	0.289
Vasopressor use^d^	2.58 (2.3–2.94)	2.55 (2.24–2.90)	2.60 (2.33–2.91)	2.45 (2.09–2.87)	<0.0001	0.943
Use of intra–arterial vasodilator therapy^e^	0.48 (0.41–0.56)	0.51 (0.45–0.58)	0.47 (0.42–0.52)	0.50 (0.42–0.59)	<0.0001	0.725
Cerebral angioplasty	0.77 (0.64–0.94)	0.71 (0.58–0.86)	0.81 (0.68–0.96)	0.71 (0.54–0.92)	0.009	0.715

*SOI, Severity of Illness. ^a^Defined as any medical comorbidity prior to ictus. ^b^Defined by the All Patients Refined Diagnosis Related Groups (APR-DRG) disease-specific risk-of-mortality severity index; reference group: minor SOI. ^c^Defined as any hospital complication during admission. ^d^Included intravenous norepinephrine, epinephrine, dopamine, vasopressin, dobutamine, milnirone. ^e^Included intra-arterial nicardipine, verapamil, milnirone, papaverine*.

Factors independently associated with reduced odds for poor discharge outcome included subsequent years of admission (2014-18 vs. 2009-13: aOR, 0.69, 95% CI, 0.66–0.71), aneurysmal repair by clipping (aOR, 0.89, 95% CI, 0.85–0.93), nimodipine use (0.44, 95% CI, 0.41–0.47) and intra-arterial vasodilator rescue therapy (aOR, 0.66, 95% CI, 0.64–0.72), whereas factors increasing odds for poor outcome included higher age (aOR per y, 1.045, 95% CI, 1.044–1.046), presence of a comorbidity flag (aOR, 1.21, 95% CI, 1.15–1.27), higher APR-DRG admission SOI (extreme vs. minor: aOR, 20.64, 95% CI, 18.52–23.00), any hospital complication flag (aOR, 3.05, 95% CI, 2.94–3.15), vasopressor use (aOR, 1.74, 95% CI, 1.64–1.85) and hospital US region MW (MW vs. NE: aOR 1.13, 95% CI, 1.09–1.18) and W (W vs. NE: aOR, 1.09, 95% CI, 1.04–1.14) (c-statistic for discharge outcome model: 0.809). Regional variability in discharge outcome predictors with corresponding aOR and 95% CI by each US region are shown in Table 5. Regional interaction effect revealed that factors contributing to regional variability in poor discharge outcomes included variability in aneurysm repair, admission SOI and nimodipine use.

**Table 5:** Multivariable logistic regression models predicting poor discharge outcome (NIS-SOM) by US region.

**Variable**	**Adjusted odds ratio estimates (95% confidence intervals)**				***P*** **value**	
Year of admission: 2014–2018 versus 2009–2013	0.69 (0.66–0.71)				<0.0001	
	**Northeast**	**Midwest**	**South**	**West**	**Main effect**	**Regional interaction Effect**
Aneurysm repair						0.001
Endovascular coiling	0.96 (0.87–1.07)	1.14 (1.03–1.27)	0.84 (0.77–0.92)	0.96 (0.84–1.11)	0.605	
Surgical clipping	0.96 (0.87–1.07)	0.90 (0.82–0.98)	0.88 (0.81–0.95)	0.82 (0.74–0.90)		
						
Age, per year	1.05 (1.04–1.05)	1.05 (1.04–1.05)	1.05 (1.04–1.05)	1.04 (1.04–1.05)	<0.0001	0.171
Presence of a comorbidity flag^a^	1.181(1.07–1.30)	1.28 (1.16–1.42)	1.16 (1.06–1.26)	1.28 (1.14–1.45)	0.001	0.305
						
Severity of illness (SOI) on admission^b^						
Moderate SOI	1.91 (1.54–2.4)	2.02 (1.60–2.54)	1.91 (1.56–2.34)	1.70 (1.24–2.33)	<0.0001	<0.0001
Major SOI	3.05 (2.50–3.73)	3.98 (3.21–4.93)	3.28 (2.73–3.96)	3.34 (2.50–4.47)		
Extreme SOI	18.49(15.1–22.7)	22.6 (18.2–28.1)	21.8 (18.1–26.3)	18.7 (13.9–25.2)		
						
Presence of a complication flag^c^	2.98 (2.78–3.20)	3.08(2.878–3.30)	3.08 (2.90–3.27)	3.01 (2.76–3.28)	<0.0001	0.886
						
Nimodipine use	0.43 (0.38–0.50)	0.48 (0.42–0.55)	0.39 (0.34–0.43)	0.49 (0.42–0.58)	<0.0001	0.025
Vasopressor use^d^	1.63 (1.44–1.84)	1.75 (1.55–1.97)	1.81 (1.63–2.01)	1.79 (1.54–2.08)	<0.0001	0.615
Use of intra-arterial vasodilator therapy^e^	0.65 (0.57–0.74)	0.70 (0.63–0.79)	0.66 (0.60–0.73)	0.65 (0.56–0.75)	<0.0001	0.771

*SOI, Severity of Illness. ^a^Defined as any medical comorbidity prior to ictus. ^b^Defined by the All Patients Refined Diagnosis Related Groups (APR-DRG) disease-specific risk of mortality severity index; reference group: Minor SOI. ^c^Defined as any hospital complication during admission. ^d^Included intravenous norepinephrine, epinephrine, dopamine, vasopressin, dobutamine, milnirone. ^e^Included intra-arterial nicardipine, verapamil, milnirone, papaverine*.

## Discussion

In this 10-year retrospective cross-sectional cohort study of 109,034 non-traumatic SAH patients, we found significant regional variability in patient characteristics and hospital interventions across the US. While there was only a modest variability in hospital outcomes, hospital complications also had significant variability across the US. Factors contributing to variability in discharge outcomes included variability in admission SOI, nimodipine use and aneurysm repair. Our study also demonstrated a continued progressive improvement in hospital mortality and discharge outcome every year, indicating an overall improvement in SAH care across the US, consistent with prior studies (6).

### Strengths and Limitations

Strengths of this study include data from a large cohort of non-traumatic SAH patients admitted to majority of academic institutions in the US. Such a very large cohort of SAH patients from multiple academic centers and affiliated hospitals across the US over a 10-year period, with updated data through 2018, has allowed us to provide insights into more recent trends in care practices, complications and outcomes across the nation. The Vizient CDB comprise detailed information on hospital care and hospital charges associated with resources used. In addition, the risk adjustment model available in the database has been validated and commonly used for comparison of institutions and estimations of quality of care delivered (10, 15, 17–21), providing a unique insight into SAH care in the country.

There are several limitations of this study which need to be highlighted. First, it is important to acknowledge that our cohort had lower proportions of patients who underwent aneurysmal clipping/coiling, than one would expect in a cohort of non-traumatic SAH patients. There are several reasons that may have contributed to this. The use of ICD codes to identify patients may be associated with reporting and misclassification biases, as ICD codes rely on appropriate coding by hospital providers. This may have led to traumatic and other non-aneurysmal SAH cases being wrongly classified into this cohort, increasing the denominator. In addition, billing codes were used for identification of aneurysmal repair interventions. These may be subject to under-, or over-coding and may also have led to a misclassification bias, leading to lower proportions of endovascular coiling and surgical clipping. To mitigate this, we used validated ICD codes and procedural codes. However, we do hypothesize that the lower proportions of coiling/clipping procedures in this cohort may, at least in part, truly reflect SAH care across the country, given that aneurysmal repair was independently associated with improved outcomes in multivariable models. Notably, in this cohort patients with a higher clinical severity-of-illness index (SOI) on admission were less likely to receive aneurysmal repair interventions. Thirty one percent with minor SOI underwent aneurysmal repair vs. only 9% with major/extreme SOI, and majority of this cohort were classified as major and extreme SOI patients. This may be due to the withholding of aneurysmal repair among higher SAH grade patients, which has been considered a common practice in many centers (30–32).

Other limitations include identification of DCI as complication, given that there is no valid ICD code and DCI was defined by combining ICD codes for vasospasm and ischemic stroke with SAH. Additionally, we used mortality, discharge disposition and procedural codes for tracheostomy/gastrostomy to define poor discharge-outcome (NIS-SOM), as the database did not include functional outcome measures such as mRS or Glasgow outcome scale. Although this is not ideal, definitive end-points such as mortality, discharge disposition and procedural codes are not often impacted by misclassification bias, due to their association with billing. In addition, NIS-SOM is an externally validated outcome measure with a strong correlation and high agreement with poor mRS defined as mRS 4–6 (27). Nonetheless, we acknowledge that discharge outcomes are less meaningful when compared to longer-term outcomes such as 90 or 180-day outcomes, limiting our understanding of the true impact of variability in care on recovery after SAH. Moreover, regional variation in availability of different types of post-acute care discharge facilities, regional socioeconomic disparities and geographical impediments leading to delay in access to advanced SAH care, may have also confounded this study findings, which was not accounted for in this study. Finally, the database also did not include information on well-known SAH severity measures such as Hunt-Hess or WFNS grades, although the APR-DRG admission SOI risk-of-mortality index is disease-specific (25) and has also been shown previously to be a good predictor of functional outcome and mortality after SAH (27). We could not account for unmeasured confounders such as location of the aneurysm, timing of aneurysm treatment, granular information regarding neuroimaging characteristics of SAH, dosing/frequency and duration of medications used as these were not available in the administrative data. Hence, more detailed analyses were not feasible. Most importantly, ICD-9 and 10 codes could not differentiate non-aneurysmal from aneurysmal SAH and thus our cohort of patients included all non-traumatic SAH patients, regardless of etiology. We acknowledge that variability in care practices and outcomes may be related to variability in the geographic distribution of aneurysmal vs. non-aneurysmal SAH patients, however the goal of this study was to assess hospital-care across all SAH patients, regardless of etiology.

### Implications of the Study

While variability in outcome has been studied previously (33–35), this is the first study evaluating regional variability in care among SAH patients using patient-level data in the US. Reasons for variability in care remain unclear, but may be related to limited Class I data guiding therapy in SAH as well as significant variability in access to specialized care. A prior study showed that patients admitted to low-volume SAH centers have worse outcomes when compared to high volume centers (36). The W had a lower proportion of high-volume SAH centers compared to other regions in our cohort. This may explain the higher use of aneurysmal surgical clipping in the W, and consequent complications, particularly DCI, seizures and CNS infections. High-volume SAH centers are most often designated comprehensive stroke centers, and thus, have round-the-clock availability of endovascular neurointerventionalists, providing easy access to endovascular coiling. There is a national shortage of neurointerventionalists, with most US centers unable to meet the required neurointerventional procedure volume to ensure adequate operator experience (37). In a recent study, there was significant geographic disparity in proximity to certified stroke centers with higher disparity in the W compared to other regions (38). Majority of the central hub stroke-centers in the W are located in the more populous states of California, Oregon and Washington, with significant geographic impediments delaying access to advanced care, including prolonged travel times due to mountain travel, long distances, and weather impediments. This hypothesis needs further exploration including evaluation of population-to-neurointerventionalist and population-to-comprehensive-stroke-center ratios in different US regions and their relation to SAH care and outcomes. A prior study showed that higher population-to-neurosurgeon ratio and higher per-capita GDP were associated with lower mortality and better neurological outcomes in SAH likely due to centralized care and better resource availability (35).

Despite variability in care, we found only a modest variability in hospital mortality and discharge outcomes across US geographic regions. This is largely consistent with prior literature that found no significant difference in outcomes in SAH patients between countries and continents (11, 33), although there were differences in outcome between centers (33), which may be related to variability in experience in management of SAH patients. Our study, however, found significant regional variability in hospital complications, length of stay, likelihood of discharge to home/acute rehabilitation and health-care expenditure, which may be an influence of differences in care practices. For example, the W had higher incidence of DCI, seizures and systemic infections which are more commonly associated with surgical clipping after SAH (39). Higher length of stay in the W may be due to higher use of surgical clipping and its consequent complications. Similarly, lower prevalence of cardio-pulmonary complications in the NE, may be due to lower use of albumin and vasopressors as well as variability in fluid therapy (40), although we did not study volume and type of intravenous crystalloids used. Baseline patient characteristics, such as admission SOI and co-morbidities may also contribute to the variability in complications. For example, the S had a higher proportion of patients with extreme admission SOI, which may explain higher prevalence of cerebral edema, hydrocephalus and consequently, respiratory failure and need for tracheostomy. Vasopressor use was also higher in the S and this is associated with higher risk for global cerebral edema (41).

The most important factor associated with hospital outcome included SOI upon admission, a surrogate marker for SAH severity (27). This is consistent with prior studies which have shown that SAH severity grade remains the most important predictor of outcome (42). Patients with extreme SOI had an 11–22 fold higher risk for death and poor discharge outcome in our study. Consequently, admission SOI was also an independent factor driving regional variability in outcomes. Other patient characteristics associated with variability in outcome included age, hospital complications and co-morbidities. Presence of a co-morbidity reduced odds for hospital mortality but increased odds for poor discharge outcome, which may be a consequence of survival in a poor functional state. A prior study evaluating impact of co-morbidities on SAH outcome, however, did not find any association with outcome after SAH (43).

Among hospital interventions, aneurysm repair, nimodipine use, vasopressor use and EVT for DCI were associated with outcome. The benefits of early aneurysm repair have been known for decades (3, 44). Our study also demonstrated that aneurysm repair by surgical clipping or endovascular coiling significantly reduced odds for hospital mortality.

Nimodipine use was associated with reduction in mortality and poor discharge outcome, consistent with prior studies (45–47). Moreover, variability in nimodipine use also contributed to variability in discharge outcomes across US regions. In contrast, vasopressor use was associated with higher odds for death and poor discharge outcome. We were unable to delineate whether vasopressors were used for induced hypertension to treat DCI or to treat shock and hypotension. It is possible that the inverse relationship between vasopressor use and outcome may be due to higher incidence of hypotension and other complications among patients that needed vasopressors. However, even after adjusting for admission SOI and any complication flag, vasopressor use was independently associated with poor outcome in multivariable models. This is the first study showing worse outcomes with vasopressor use in SAH, however, prior studies have shown higher incidence of neurocardiogenic injury and cerebral edema in SAH patients receiving vasopressors (41, 48).

EVT for DCI, particularly intra-arterial vasodilator therapy, significantly reduced odds for hospital mortality and poor discharge outcome. There are no randomized trials that have assessed outcome benefit with EVT in DCI, however a large meta-analysis of 55 smaller observational studies showed significant radiographic benefit with only a modest clinical benefit (49). This is the first large multicenter study of more than hundred thousand patients that has shown significant improvement in discharge outcomes with EVT for DCI. Another recent study of 1,000 patients showed that early and more frequent EVT increased odds for favorable outcome compared to a more restrictive strategy (50) in DCI.

Other factors of significance that warrant discussion include variability in management of post-SAH pain and headache (51) as well as elevated intracranial pressure (ICP) (52). Our ability to study these was limited due to limited availability of more granular inpatient data in the Vizient data-base, including patient-reported pain, behavioral pain scores, ICP recordings and related interventions. We did find variability in the use of extra-ventricular drains for ICP management, with higher use in S and W (24%) vs. NE and MW (21%). It is however likely, that there is significant variation in management of these SAH-related complications, including in the use of steroids and hyperosmolar therapy.

## Conclusions and Future Research

Our study suggests that significant regional variability in care of SAH patients exists in the US. In addition, our data demonstrates a significant association between such variability and a modest worsening in hospital outcomes and complications. Our findings need further confirmation in well-conducted prospective observational studies, which should incorporate detailed information regarding SAH severity, imaging data, and long-term clinical outcomes. Such prospective, observational studies will afford the opportunity for comparative-effectiveness research (CER) analyses to determine which treatments/interventions may have a significant effect on long-term outcomes and overall resource use and cost.

## Data Availability Statement

The data analyzed in this study is subject to the following licenses/restrictions: The dataset includes individual de-identified patient-level data from more than 95% of academic centers in the US that participate in the Vizient Clinical DataBase. The data is collected and stored by Vizient Inc. and can be obtained by providing a written research proposal directly to Vizient Inc. Requests to access these datasets should be directed to clinicalanalytics@vizientinc.com.

## Ethics Statement

The studies involving human participants were reviewed and approved by Johns Hopkins Institutional Review Board. Written informed consent for participation was not required for this study in accordance with the national legislation and the institutional requirements.

## Author Contributions

VS participated in conceptualizing study, data collection, planning data analysis, interpretation and presentation of results, and wrote the first draft of the manuscript. SK, RD, and EC participated in data collection and review and editing of manuscript. AH performed data collection, data analysis, participated in interpretation and presentation of results, and review and editing of manuscript. SH participated in data analysis, interpretation and presentation of results, and review and editing of manuscript. JS conceptualized the study, formulated hypothesis, participated in data collection, analysis, and review and editing of manuscript. All authors contributed to the article and approved the submitted version.

## Conflict of Interest

AH and SH are employed by Center for Advanced Analytics and Informatics, Vizient, Inc. None of the authors had any financial relationships except JS, who is the Chair of the DSMB for the INTREPID Study funded by BARD, member of the CEC for the REACT Study funded by Idorsia, member of the Editorial Board of Stroke, and an ex-officio member of the Board of Directors of the Neurocritical Care Society. The remaining authors declare that the research was conducted in the absence of any commercial or financial relationships that could be construed as a potential conflict of interest.

## Publisher's Note

All claims expressed in this article are solely those of the authors and do not necessarily represent those of their affiliated organizations, or those of the publisher, the editors and the reviewers. Any product that may be evaluated in this article, or claim that may be made by its manufacturer, is not guaranteed or endorsed by the publisher.
