# Supplementary material for: Regional Variability in the Care and Outcomes of Subarachnoid Hemorrhage Patients in the United States
Source: Front Neurol. 2022 Jun 16;13:908609. doi: 10.3389/fneur.2022.908609 (PMC9243235; doi:10.3389/fneur.2022.908609)
Supplement: Supplementary file 1 [file Table_1.DOCX]

**ICD Codes for non-traumatic SAH Case Definition:**

| ICD Set | Code | Description | Category |
| --- | --- | --- | --- |
| 10 | I60 | Nontraumatic subarachnoid hemorrhage | SAH |
| 10 | I600 | Nontraumatic subarachnoid hemorrhage from carotid siphon and bifurcation | SAH |
| 10 | I6000 | Nontraumatic subarachnoid hemorrhage from unspecified carotid siphon and bifurcation | SAH |
| 10 | I6001 | Nontraumatic subarachnoid hemorrhage from right carotid siphon and bifurcation | SAH |
| 10 | I6002 | Nontraumatic subarachnoid hemorrhage from left carotid siphon and bifurcation | SAH |
| 10 | I601 | Nontraumatic subarachnoid hemorrhage from middle cerebral artery | SAH |
| 10 | I6010 | Nontraumatic subarachnoid hemorrhage from unspecified middle cerebral artery | SAH |
| 10 | I6011 | Nontraumatic subarachnoid hemorrhage from right middle cerebral artery | SAH |
| 10 | I6012 | Nontraumatic subarachnoid hemorrhage from left middle cerebral artery | SAH |
| 10 | I602 | Nontraumatic subarachnoid hemorrhage from anterior communicating artery | SAH |
| 10 | I6020 | Nontraumatic subarachnoid hemorrhage from unspecified anterior communicating artery | SAH |
| 10 | I6021 | Nontraumatic subarachnoid hemorrhage from right anterior communicating artery | SAH |
| 10 | I6022 | Nontraumatic subarachnoid hemorrhage from left anterior communicating artery | SAH |
| 10 | I603 | Nontraumatic subarachnoid hemorrhage from posterior communicating artery | SAH |
| 10 | I6030 | Nontraumatic subarachnoid hemorrhage from unspecified posterior communicating artery | SAH |
| 10 | I6031 | Nontraumatic subarachnoid hemorrhage from right posterior communicating artery | SAH |
| 10 | I6032 | Nontraumatic subarachnoid hemorrhage from left posterior communicating artery | SAH |
| 10 | I604 | Nontraumatic subarachnoid hemorrhage from basilar artery | SAH |
| 10 | I605 | Nontraumatic subarachnoid hemorrhage from vertebral artery | SAH |
| 10 | I6050 | Nontraumatic subarachnoid hemorrhage from unspecified vertebral artery | SAH |
| 10 | I6051 | Nontraumatic subarachnoid hemorrhage from right vertebral artery | SAH |
| 10 | I6052 | Nontraumatic subarachnoid hemorrhage from left vertebral artery | SAH |
| 10 | I606 | Nontraumatic subarachnoid hemorrhage from other intracranial arteries | SAH |
| 10 | I607 | Nontraumatic subarachnoid hemorrhage from unspecified intracranial artery | SAH |
| 10 | I608 | Other nontraumatic subarachnoid hemorrhage | SAH |
| 10 | I609 | Nontraumatic subarachnoid hemorrhage, unspecified | SAH |
| 9 | 430 | Subarachnoid hemorrhage | SAH |

**Delayed Cerebral Ischemia Case Definition:**

| SAH + DCI symptom |
| --- |
| SAH only |
| SAH + ischemia |
| SAH + vasospasm |
| SAH + cerebral infarction |
| SAH + sequelae (SAH, Infarction or non-specific) |
| SAH + ischemia + vasospasm |
| SAH + ischemia + cerebral infarction |
| SAH + vasospasm + cerebral infarction |
| SAH + ischemia + sequelae (SAH, infarction or non-specific) |
| SAH + vasospasm + sequelae (SAH, infarction or non-specific) |
| SAH + cerebral infarction + sequelae (SAH, infarction or non-specific) |
| SAH + ischemia + vasospasm + cerebral infarction |
| SAH + ischemia + vasospasm + sequelae |
| SAH + ischemia + cerebral infarction + sequelae |
| SAH + vasospasm + cerebral infarction + sequelae |
| SAH + ischemia + vasospasm + cerebral infarction +sequelae |

**ICD codes used to identify ischemia, vasospasm, cerebral infarction, sequelae:**

| 10 | I63 | Cerebral infarction | infarction |
| --- | --- | --- | --- |
| 10 | I630 | Cerebral infarction due to thrombosis of precerebral arteries | infarction |
| 10 | I6300 | Cerebral infarction due to thrombosis of unspecified precerebral artery | infarction |
| 10 | I6301 | Cerebral infarction due to thrombosis of vertebral artery | infarction |
| 10 | I63011 | Cerebral infarction due to thrombosis of right vertebral artery | infarction |
| 10 | I63012 | Cerebral infarction due to thrombosis of left vertebral artery | infarction |
| 10 | I63019 | Cerebral infarction due to thrombosis of unspecified vertebral artery | infarction |
| 10 | I6302 | Cerebral infarction due to thrombosis of basilar artery | infarction |
| 10 | I6303 | Cerebral infarction due to thrombosis of carotid artery | infarction |
| 10 | I63031 | Cerebral infarction due to thrombosis of right carotid artery | infarction |
| 10 | I63032 | Cerebral infarction due to thrombosis of left carotid artery | infarction |
| 10 | I63039 | Cerebral infarction due to thrombosis of unspecified carotid artery | infarction |
| 10 | I6309 | Cerebral infarction due to thrombosis of other precerebral artery | infarction |
| 10 | I631 | Cerebral infarction due to embolism of precerebral arteries | infarction |
| 10 | I6310 | Cerebral infarction due to embolism of unspecified precerebral artery | infarction |
| 10 | I6311 | Cerebral infarction due to embolism of vertebral artery | infarction |
| 10 | I63111 | Cerebral infarction due to embolism of right vertebral artery | infarction |
| 10 | I63112 | Cerebral infarction due to embolism of left vertebral artery | infarction |
| 10 | I63119 | Cerebral infarction due to embolism of unspecified vertebral artery | infarction |
| 10 | I6312 | Cerebral infarction due to embolism of basilar artery | infarction |
| 10 | I6313 | Cerebral infarction due to embolism of carotid artery | infarction |
| 10 | I63131 | Cerebral infarction due to embolism of right carotid artery | infarction |
| 10 | I63132 | Cerebral infarction due to embolism of left carotid artery | infarction |
| 10 | I63139 | Cerebral infarction due to embolism of unspecified carotid artery | infarction |
| 10 | I6319 | Cerebral infarction due to embolism of other precerebral artery | infarction |
| 10 | I632 | Cerebral infarction due to unspecified occlusion or stenosis of precerebral arteries | infarction |
| 10 | I6320 | Cerebral infarction due to unspecified occlusion or stenosis of unspecified precerebral arteries | infarction |
| 10 | I6321 | Cerebral infarction due to unspecified occlusion or stenosis of vertebral arteries | infarction |
| 10 | I63211 | Cerebral infarction due to unspecified occlusion or stenosis of right vertebral arteries | infarction |
| 10 | I63212 | Cerebral infarction due to unspecified occlusion or stenosis of left vertebral arteries | infarction |
| 10 | I63219 | Cerebral infarction due to unspecified occlusion or stenosis of unspecified vertebral arteries | infarction |
| 10 | I6322 | Cerebral infarction due to unspecified occlusion or stenosis of basilar arteries | infarction |
| 10 | I6323 | Cerebral infarction due to unspecified occlusion or stenosis of carotid arteries | infarction |
| 10 | I63231 | Cerebral infarction due to unspecified occlusion or stenosis of right carotid arteries | infarction |
| 10 | I63232 | Cerebral infarction due to unspecified occlusion or stenosis of left carotid arteries | infarction |
| 10 | I63239 | Cerebral infarction due to unspecified occlusion or stenosis of unspecified carotid arteries | infarction |
| 10 | I6329 | Cerebral infarction due to unspecified occlusion or stenosis of other precerebral arteries | infarction |
| 10 | I633 | Cerebral infarction due to thrombosis of cerebral arteries | infarction |
| 10 | I6330 | Cerebral infarction due to thrombosis of unspecified cerebral artery | infarction |
| 10 | I6331 | Cerebral infarction due to thrombosis of middle cerebral artery | infarction |
| 10 | I63311 | Cerebral infarction due to thrombosis of right middle cerebral artery | infarction |
| 10 | I63312 | Cerebral infarction due to thrombosis of left middle cerebral artery | infarction |
| 10 | I63319 | Cerebral infarction due to thrombosis of unspecified middle cerebral artery | infarction |
| 10 | I6332 | Cerebral infarction due to thrombosis of anterior cerebral artery | infarction |
| 10 | I63321 | Cerebral infarction due to thrombosis of right anterior cerebral artery | infarction |
| 10 | I63322 | Cerebral infarction due to thrombosis of left anterior cerebral artery | infarction |
| 10 | I63329 | Cerebral infarction due to thrombosis of unspecified anterior cerebral artery | infarction |
| 10 | I6333 | Cerebral infarction due to thrombosis of posterior cerebral artery | infarction |
| 10 | I63331 | Cerebral infarction due to thrombosis of right posterior cerebral artery | infarction |
| 10 | I63332 | Cerebral infarction due to thrombosis of left posterior cerebral artery | infarction |
| 10 | I63339 | Cerebral infarction due to thrombosis of unspecified posterior cerebral artery | infarction |
| 10 | I6334 | Cerebral infarction due to thrombosis of cerebellar artery | infarction |
| 10 | I63341 | Cerebral infarction due to thrombosis of right cerebellar artery | infarction |
| 10 | I63342 | Cerebral infarction due to thrombosis of left cerebellar artery | infarction |
| 10 | I63349 | Cerebral infarction due to thrombosis of unspecified cerebellar artery | infarction |
| 10 | I6339 | Cerebral infarction due to thrombosis of other cerebral artery | infarction |
| 10 | I634 | Cerebral infarction due to embolism of cerebral arteries | infarction |
| 10 | I6340 | Cerebral infarction due to embolism of unspecified cerebral artery | infarction |
| 10 | I6341 | Cerebral infarction due to embolism of middle cerebral artery | infarction |
| 10 | I63411 | Cerebral infarction due to embolism of right middle cerebral artery | infarction |
| 10 | I63412 | Cerebral infarction due to embolism of left middle cerebral artery | infarction |
| 10 | I63419 | Cerebral infarction due to embolism of unspecified middle cerebral artery | infarction |
| 10 | I6342 | Cerebral infarction due to embolism of anterior cerebral artery | infarction |
| 10 | I63421 | Cerebral infarction due to embolism of right anterior cerebral artery | infarction |
| 10 | I63422 | Cerebral infarction due to embolism of left anterior cerebral artery | infarction |
| 10 | I63429 | Cerebral infarction due to embolism of unspecified anterior cerebral artery | infarction |
| 10 | I6343 | Cerebral infarction due to embolism of posterior cerebral artery | infarction |
| 10 | I63431 | Cerebral infarction due to embolism of right posterior cerebral artery | infarction |
| 10 | I63432 | Cerebral infarction due to embolism of left posterior cerebral artery | infarction |
| 10 | I63439 | Cerebral infarction due to embolism of unspecified posterior cerebral artery | infarction |
| 10 | I6344 | Cerebral infarction due to embolism of cerebellar artery | infarction |
| 10 | I63441 | Cerebral infarction due to embolism of right cerebellar artery | infarction |
| 10 | I63442 | Cerebral infarction due to embolism of left cerebellar artery | infarction |
| 10 | I63449 | Cerebral infarction due to embolism of unspecified cerebellar artery | infarction |
| 10 | I6349 | Cerebral infarction due to embolism of other cerebral artery | infarction |
| 10 | I635 | Cerebral infarction due to unspecified occlusion or stenosis of cerebral arteries | infarction |
| 10 | I6350 | Cerebral infarction due to unspecified occlusion or stenosis of unspecified cerebral artery | infarction |
| 10 | I6351 | Cerebral infarction due to unspecified occlusion or stenosis of middle cerebral artery | infarction |
| 10 | I63511 | Cerebral infarction due to unspecified occlusion or stenosis of right middle cerebral artery | infarction |
| 10 | I63512 | Cerebral infarction due to unspecified occlusion or stenosis of left middle cerebral artery | infarction |
| 10 | I63519 | Cerebral infarction due to unspecified occlusion or stenosis of unspecified middle cerebral artery | infarction |
| 10 | I6352 | Cerebral infarction due to unspecified occlusion or stenosis of anterior cerebral artery | infarction |
| 10 | I63521 | Cerebral infarction due to unspecified occlusion or stenosis of right anterior cerebral artery | infarction |
| 10 | I63522 | Cerebral infarction due to unspecified occlusion or stenosis of left anterior cerebral artery | infarction |
| 10 | I63529 | Cerebral infarction due to unspecified occlusion or stenosis of unspecified anterior cerebral artery | infarction |
| 10 | I6353 | Cerebral infarction due to unspecified occlusion or stenosis of posterior cerebral artery | infarction |
| 10 | I63531 | Cerebral infarction due to unspecified occlusion or stenosis of right posterior cerebral artery | infarction |
| 10 | I63532 | Cerebral infarction due to unspecified occlusion or stenosis of left posterior cerebral artery | infarction |
| 10 | I63539 | Cerebral infarction due to unspecified occlusion or stenosis of unspecified posterior cerebral artery | infarction |
| 10 | I6354 | Cerebral infarction due to unspecified occlusion or stenosis of cerebellar artery | infarction |
| 10 | I63541 | Cerebral infarction due to unspecified occlusion or stenosis of right cerebellar artery | infarction |
| 10 | I63542 | Cerebral infarction due to unspecified occlusion or stenosis of left cerebellar artery | infarction |
| 10 | I63549 | Cerebral infarction due to unspecified occlusion or stenosis of unspecified cerebellar artery | infarction |
| 10 | I6359 | Cerebral infarction due to unspecified occlusion or stenosis of other cerebral artery | infarction |
| 10 | I636 | Cerebral infarction due to cerebral venous thrombosis, nonpyogenic | infarction |
| 10 | I638 | Other cerebral infarction | infarction |
| 10 | I639 | Cerebral infarction, unspecified | infarction |
| 10 | I6782 | Cerebral ischemia | ischemia |
| 10 | I6784 | Cerebral vasospasm and vasoconstriction | vasospasm |
| 10 | I67841 | Reversible cerebrovascular vasoconstriction syndrome | vasospasm |
| 10 | I67848 | Other cerebrovascular vasospasm and vasoconstriction | vasospasm |
| 10 | I69 | Sequelae of cerebrovascular disease | SAH sequelae |
| 10 | I690 | Sequelae of nontraumatic subarachnoid hemorrhage | SAH sequelae |
| 10 | I6900 | Unspecified sequelae of nontraumatic subarachnoid hemorrhage | SAH sequelae |
| 10 | I6901 | Cognitive deficits following nontraumatic subarachnoid hemorrhage | SAH sequelae |
| 10 | I6902 | Speech and language deficits following nontraumatic subarachnoid hemorrhage | SAH sequelae |
| 10 | I69020 | Aphasia following nontraumatic subarachnoid hemorrhage | SAH sequelae |
| 10 | I69021 | Dysphasia following nontraumatic subarachnoid hemorrhage | SAH sequelae |
| 10 | I69022 | Dysarthria following nontraumatic subarachnoid hemorrhage | SAH sequelae |
| 10 | I69023 | Fluency disorder following nontraumatic subarachnoid hemorrhage | SAH sequelae |
| 10 | I69028 | Other speech and language deficits following nontraumatic subarachnoid hemorrhage | SAH sequelae |
| 10 | I6903 | Monoplegia of upper limb following nontraumatic subarachnoid hemorrhage | SAH sequelae |
| 10 | I69031 | Monoplegia of upper limb following nontraumatic subarachnoid hemorrhage affecting right dominant side | SAH sequelae |
| 10 | I69032 | Monoplegia of upper limb following nontraumatic subarachnoid hemorrhage affecting left dominant side | SAH sequelae |
| 10 | I69033 | Monoplegia of upper limb following nontraumatic subarachnoid hemorrhage affecting right non-dominant side | SAH sequelae |
| 10 | I69034 | Monoplegia of upper limb following nontraumatic subarachnoid hemorrhage affecting left non-dominant side | SAH sequelae |
| 10 | I69039 | Monoplegia of upper limb following nontraumatic subarachnoid hemorrhage affecting unspecified side | SAH sequelae |
| 10 | I6904 | Monoplegia of lower limb following nontraumatic subarachnoid hemorrhage | SAH sequelae |
| 10 | I69041 | Monoplegia of lower limb following nontraumatic subarachnoid hemorrhage affecting right dominant side | SAH sequelae |
| 10 | I69042 | Monoplegia of lower limb following nontraumatic subarachnoid hemorrhage affecting left dominant side | SAH sequelae |
| 10 | I69043 | Monoplegia of lower limb following nontraumatic subarachnoid hemorrhage affecting right non-dominant side | SAH sequelae |
| 10 | I69044 | Monoplegia of lower limb following nontraumatic subarachnoid hemorrhage affecting left non-dominant side | SAH sequelae |
| 10 | I69049 | Monoplegia of lower limb following nontraumatic subarachnoid hemorrhage affecting unspecified side | SAH sequelae |
| 10 | I6905 | Hemiplegia and hemiparesis following nontraumatic subarachnoid hemorrhage | SAH sequelae |
| 10 | I69051 | Hemiplegia and hemiparesis following nontraumatic subarachnoid hemorrhage affecting right dominant side | SAH sequelae |
| 10 | I69052 | Hemiplegia and hemiparesis following nontraumatic subarachnoid hemorrhage affecting left dominant side | SAH sequelae |
| 10 | I69053 | Hemiplegia and hemiparesis following nontraumatic subarachnoid hemorrhage affecting right non-dominant side | SAH sequelae |
| 10 | I69054 | Hemiplegia and hemiparesis following nontraumatic subarachnoid hemorrhage affecting left non-dominant side | SAH sequelae |
| 10 | I69059 | Hemiplegia and hemiparesis following nontraumatic subarachnoid hemorrhage affecting unspecified side | SAH sequelae |
| 10 | I6906 | Other paralytic syndrome following nontraumatic subarachnoid hemorrhage | SAH sequelae |
| 10 | I69061 | Other paralytic syndrome following nontraumatic subarachnoid hemorrhage affecting right dominant side | SAH sequelae |
| 10 | I69062 | Other paralytic syndrome following nontraumatic subarachnoid hemorrhage affecting left dominant side | SAH sequelae |
| 10 | I69063 | Other paralytic syndrome following nontraumatic subarachnoid hemorrhage affecting right non-dominant side | SAH sequelae |
| 10 | I69064 | Other paralytic syndrome following nontraumatic subarachnoid hemorrhage affecting left non-dominant side | SAH sequelae |
| 10 | I69065 | Other paralytic syndrome following nontraumatic subarachnoid hemorrhage, bilateral | SAH sequelae |
| 10 | I69069 | Other paralytic syndrome following nontraumatic subarachnoid hemorrhage affecting unspecified side | SAH sequelae |
| 10 | I6909 | Other sequelae of nontraumatic subarachnoid hemorrhage | SAH sequelae |
| 10 | I69090 | Apraxia following nontraumatic subarachnoid hemorrhage | SAH sequelae |
| 10 | I69091 | Dysphagia following nontraumatic subarachnoid hemorrhage | SAH sequelae |
| 10 | I69092 | Facial weakness following nontraumatic subarachnoid hemorrhage | SAH sequelae |
| 10 | I69093 | Ataxia following nontraumatic subarachnoid hemorrhage | SAH sequelae |
| 10 | I69098 | Other sequelae following nontraumatic subarachnoid hemorrhage | SAH sequelae |
| 10 | I693 | Sequelae of cerebral infarction | infarcation sequelae |
| 10 | I6930 | Unspecified sequelae of cerebral infarction | infarcation sequelae |
| 10 | I6931 | Cognitive deficits following cerebral infarction | infarcation sequelae |
| 10 | I6932 | Speech and language deficits following cerebral infarction | infarcation sequelae |
| 10 | I69320 | Aphasia following cerebral infarction | infarcation sequelae |
| 10 | I69321 | Dysphasia following cerebral infarction | infarcation sequelae |
| 10 | I69322 | Dysarthria following cerebral infarction | infarcation sequelae |
| 10 | I69323 | Fluency disorder following cerebral infarction | infarcation sequelae |
| 10 | I69328 | Other speech and language deficits following cerebral infarction | infarcation sequelae |
| 10 | I6933 | Monoplegia of upper limb following cerebral infarction | infarcation sequelae |
| 10 | I69331 | Monoplegia of upper limb following cerebral infarction affecting right dominant side | infarcation sequelae |
| 10 | I69332 | Monoplegia of upper limb following cerebral infarction affecting left dominant side | infarcation sequelae |
| 10 | I69333 | Monoplegia of upper limb following cerebral infarction affecting right non-dominant side | infarcation sequelae |
| 10 | I69334 | Monoplegia of upper limb following cerebral infarction affecting left non-dominant side | infarcation sequelae |
| 10 | I69339 | Monoplegia of upper limb following cerebral infarction affecting unspecified side | infarcation sequelae |
| 10 | I6934 | Monoplegia of lower limb following cerebral infarction | infarcation sequelae |
| 10 | I69341 | Monoplegia of lower limb following cerebral infarction affecting right dominant side | infarcation sequelae |
| 10 | I69342 | Monoplegia of lower limb following cerebral infarction affecting left dominant side | infarcation sequelae |
| 10 | I69343 | Monoplegia of lower limb following cerebral infarction affecting right non-dominant side | infarcation sequelae |
| 10 | I69344 | Monoplegia of lower limb following cerebral infarction affecting left non-dominant side | infarcation sequelae |
| 10 | I69349 | Monoplegia of lower limb following cerebral infarction affecting unspecified side | infarcation sequelae |
| 10 | I6935 | Hemiplegia and hemiparesis following cerebral infarction | infarcation sequelae |
| 10 | I69351 | Hemiplegia and hemiparesis following cerebral infarction affecting right dominant side | infarcation sequelae |
| 10 | I69352 | Hemiplegia and hemiparesis following cerebral infarction affecting left dominant side | infarcation sequelae |
| 10 | I69353 | Hemiplegia and hemiparesis following cerebral infarction affecting right non-dominant side | infarcation sequelae |
| 10 | I69354 | Hemiplegia and hemiparesis following cerebral infarction affecting left non-dominant side | infarcation sequelae |
| 10 | I69359 | Hemiplegia and hemiparesis following cerebral infarction affecting unspecified side | infarcation sequelae |
| 10 | I6936 | Other paralytic syndrome following cerebral infarction | infarcation sequelae |
| 10 | I69361 | Other paralytic syndrome following cerebral infarction affecting right dominant side | infarcation sequelae |
| 10 | I69362 | Other paralytic syndrome following cerebral infarction affecting left dominant side | infarcation sequelae |
| 10 | I69363 | Other paralytic syndrome following cerebral infarction affecting right non-dominant side | infarcation sequelae |
| 10 | I69364 | Other paralytic syndrome following cerebral infarction affecting left non-dominant side | infarcation sequelae |
| 10 | I69365 | Other paralytic syndrome following cerebral infarction, bilateral | infarcation sequelae |
| 10 | I69369 | Other paralytic syndrome following cerebral infarction affecting unspecified side | infarcation sequelae |
| 10 | I6939 | Other sequelae of cerebral infarction | infarcation sequelae |
| 10 | I69390 | Apraxia following cerebral infarction | infarcation sequelae |
| 10 | I69391 | Dysphagia following cerebral infarction | infarcation sequelae |
| 10 | I69392 | Facial weakness following cerebral infarction | infarcation sequelae |
| 10 | I69393 | Ataxia following cerebral infarction | infarcation sequelae |
| 10 | I69398 | Other sequelae of cerebral infarction | infarcation sequelae |
| 9 | 43401 | Cerebral thrombosis with cerebral infarction | infarction |
| 9 | 43411 | Cerebral embolism with cerebral infarction | infarction |
| 9 | 43491 | Cerebral artery occlusion, unspecified with cerebral infarction | infarction |
| 9 | 43301 | Occlusion and stenosis of basilar artery with cerebral infarction | infarction |
| 9 | 43311 | Occlusion and stenosis of carotid artery with cerebral infarction | infarction |
| 9 | 43321 | Occlusion and stenosis of vertebral artery with cerebral infarction | infarction |
| 9 | 43331 | Occlusion and stenosis of multiple and bilateral precerebral arteries with cerebral infarction | infarction |
| 9 | 43381 | Occlusion and stenosis of other specified precerebral artery with cerebral infarction | infarction |
| 9 | 43391 | Occlusion and stenosis of unspecified precerebral artery with cerebral infarction | infarction |
| 9 | 4380 | Late effects of cerebrovascular disease, cognitive deficits | sequelae |
| 9 | 43810 | Late effects of cerebrovascular disease, speech and language deficit, unspecified | sequelae |
| 9 | 43811 | Late effects of cerebrovascular disease, aphasia | sequelae |
| 9 | 43812 | Late effects of cerebrovascular disease, dysphasia | sequelae |
| 9 | 43813 | Late effects of cerebrovascular disease, dysarthria | sequelae |
| 9 | 43814 | Late effects of cerebrovascular disease, fluency disorder | sequelae |
| 9 | 43819 | Late effects of cerebrovascular disease, other speech and language deficits | sequelae |
| 9 | 43820 | Late effects of cerebrovascular disease, hemiplegia affecting unspecified side | sequelae |
| 9 | 43821 | Late effects of cerebrovascular disease, hemiplegia affecting dominant side | sequelae |
| 9 | 43822 | Late effects of cerebrovascular disease, hemiplegia affecting nondominant side | sequelae |
| 9 | 43830 | Late effects of cerebrovascular disease, monoplegia of upper limb affecting unspecified side | sequelae |
| 9 | 43831 | Late effects of cerebrovascular disease, monoplegia of upper limb affecting dominant side | sequelae |
| 9 | 43832 | Late effects of cerebrovascular disease, monoplegia of upper limb affecting nondominant side | sequelae |
| 9 | 43840 | Late effects of cerebrovascular disease, monoplegia of lower limb affecting unspecified side | sequelae |
| 9 | 43841 | Late effects of cerebrovascular disease, monoplegia of lower limb affecting dominant side | sequelae |
| 9 | 43842 | Late effects of cerebrovascular disease, monoplegia of lower limb affecting nondominant side | sequelae |
| 9 | 43850 | Late effects of cerebrovascular disease, other paralytic syndrome affecting unspecified side | sequelae |
| 9 | 43851 | Late effects of cerebrovascular disease, other paralytic syndrome affecting dominant side | sequelae |
| 9 | 43852 | Late effects of cerebrovascular disease, other paralytic syndrome affecting nondominant side | sequelae |
| 9 | 43853 | Late effects of cerebrovascular disease, other paralytic syndrome, bilateral | sequelae |
| 9 | 4386 | Late effects of cerebrovascular disease, alterations of sensations | sequelae |
| 9 | 4387 | Late effects of cerebrovascular disease, disturbances of vision | sequelae |
| 9 | 43881 | Other late effects of cerebrovascular disease, apraxia | sequelae |
| 9 | 43882 | Other late effects of cerebrovascular disease, dysphagia | sequelae |
| 9 | 43883 | Other late effects of cerebrovascular disease, facial weakness | sequelae |
| 9 | 43884 | Other late effects of cerebrovascular disease, ataxia | sequelae |
| 9 | 43885 | Other late effects of cerebrovascular disease, vertigo | sequelae |
| 9 | 43889 | Other late effects of cerebrovascular disease | sequelae |
| 9 | 4389 | Unspecified late effects of cerebrovascular disease | sequelae |
| 9 | 4371 | Other generalized ischemic cerebrovascular disease | ischemia |
| 9 | 4359 | Unspecified transient cerebral ischemia | vasospasm |
| 9 | 4358 | Other specified transient cerebral ischemias | ischemia |
| 9 | 4350 | Basilar artery syndrome | ischemia |
| 9 | 4351 | Vertebral artery syndrome | ischemia |
| 9 | 4353 | Vertebrobasilar artery syndrome | ischemia |

**ICD codes for procedures/imaging/hospital complications and comorbidity case definitions:**

| ICD_Set | Code | Description | Table | Category |  |
| --- | --- | --- | --- | --- | --- |
| ICD9 | 3951 | Clipping of aneurysm | Table 3 | Aneurysm clipping |  |
| ICD10 | 03VG0CZ | 03VG0CZ - Restrict of Intracran Art with Extralum Dev, Open Approach | Table 3 | Aneurysm clipping |  |
| ICD10 | 03VG3CZ | 03VG3CZ - Restrict of Intracran Art with Extralum Dev, Perc Approach | Table 3 | Aneurysm clipping |  |
| ICD10 | 03VG4CZ | 03VG4CZ - Restrict Intracran Art w Extralum Dev, Perc Endo | Table 3 | Aneurysm clipping |  |
| ICD10 | 03VH0CZ | 03VH0CZ - Restrict of R Com Carotid with Extralum Dev, Open Approach | Table 3 | Aneurysm clipping |  |
| ICD10 | 03VH3CZ | 03VH3CZ - Restrict of R Com Carotid with Extralum Dev, Perc Approach | Table 3 | Aneurysm clipping |  |
| ICD10 | 03VH4CZ | 03VH4CZ - Restrict R Com Carotid w Extralum Dev, Perc Endo | Table 3 | Aneurysm clipping |  |
| ICD10 | 03VJ0CZ | 03VJ0CZ - Restrict of L Com Carotid with Extralum Dev, Open Approach | Table 3 | Aneurysm clipping |  |
| ICD10 | 03VJ3CZ | 03VJ3CZ - Restrict of L Com Carotid with Extralum Dev, Perc Approach | Table 3 | Aneurysm clipping |  |
| ICD10 | 03VJ4CZ | 03VJ4CZ - Restrict L Com Carotid w Extralum Dev, Perc Endo | Table 3 | Aneurysm clipping |  |
| ICD10 | 03VK0CZ | 03VK0CZ - Restrict of R Int Carotid with Extralum Dev, Open Approach | Table 3 | Aneurysm clipping |  |
| ICD10 | 03VK3CZ | 03VK3CZ - Restrict of R Int Carotid with Extralum Dev, Perc Approach | Table 3 | Aneurysm clipping |  |
| ICD10 | 03VK4CZ | 03VK4CZ - Restrict R Int Carotid w Extralum Dev, Perc Endo | Table 3 | Aneurysm clipping |  |
| ICD10 | 03VL0CZ | 03VL0CZ - Restrict of L Int Carotid with Extralum Dev, Open Approach | Table 3 | Aneurysm clipping |  |
| ICD10 | 03VL3CZ | 03VL3CZ - Restrict of L Int Carotid with Extralum Dev, Perc Approach | Table 3 | Aneurysm clipping |  |
| ICD10 | 03VL4CZ | 03VL4CZ - Restrict L Int Carotid w Extralum Dev, Perc Endo | Table 3 | Aneurysm clipping |  |
| ICD10 | 03VP0CZ | 03VP0CZ - Restriction of R Verteb Art with Extralum Dev, Open Approach | Table 3 | Aneurysm clipping |  |
| ICD10 | 03VP3CZ | 03VP3CZ - Restriction of R Verteb Art with Extralum Dev, Perc Approach | Table 3 | Aneurysm clipping |  |
| ICD10 | 03VP4CZ | 03VP4CZ - Restrict R Verteb Art w Extralum Dev, Perc Endo | Table 3 | Aneurysm clipping |  |
| ICD10 | 03VQ0CZ | 03VQ0CZ - Restriction of L Verteb Art with Extralum Dev, Open Approach | Table 3 | Aneurysm clipping |  |
| ICD10 | 03VQ3CZ | 03VQ3CZ - Restriction of L Verteb Art with Extralum Dev, Perc Approach | Table 3 | Aneurysm clipping |  |
| ICD10 | 03VQ4CZ | 03VQ4CZ - Restrict L Verteb Art w Extralum Dev, Perc Endo | Table 3 | Aneurysm clipping |  |
| ICD9 | 3972 | 3972 - ENDOV OCCL HN VESSEL | Table 3 | Aneurysm coiling |  |
| ICD10 | 03VG0ZZ | 03VG0ZZ - Restriction of Intracranial Artery, Open Approach | Table 3 | Aneurysm coiling |  |
| ICD10 | 03VG3ZZ | 03VG3ZZ - Restriction of Intracranial Artery, Percutaneous Approach | Table 3 | Aneurysm coiling |  |
| ICD10 | 03VG4ZZ | 03VG4ZZ - Restriction of Intracranial Artery, Perc Endo Approach | Table 3 | Aneurysm coiling |  |
| ICD10 | 03VH0ZZ | 03VH0ZZ - Restriction of Right Common Carotid Artery, Open Approach | Table 3 | Aneurysm coiling |  |
| ICD10 | 03VH3ZZ | 03VH3ZZ - Restriction of Right Common Carotid Artery, Perc Approach | Table 3 | Aneurysm coiling |  |
| ICD10 | 03VH4ZZ | 03VH4ZZ - Restriction of R Com Carotid, Perc Endo Approach | Table 3 | Aneurysm coiling |  |
| ICD10 | 03VJ0ZZ | 03VJ0ZZ - Restriction of Left Common Carotid Artery, Open Approach | Table 3 | Aneurysm coiling |  |
| ICD10 | 03VJ3ZZ | 03VJ3ZZ - Restriction of Left Common Carotid Artery, Perc Approach | Table 3 | Aneurysm coiling |  |
| ICD10 | 03VJ4ZZ | 03VJ4ZZ - Restriction of L Com Carotid, Perc Endo Approach | Table 3 | Aneurysm coiling |  |
| ICD10 | 03VK0ZZ | 03VK0ZZ - Restriction of Right Internal Carotid Artery, Open Approach | Table 3 | Aneurysm coiling |  |
| ICD10 | 03VK3ZZ | 03VK3ZZ - Restriction of Right Internal Carotid Artery, Perc Approach | Table 3 | Aneurysm coiling |  |
| ICD10 | 03VK4ZZ | 03VK4ZZ - Restriction of R Int Carotid, Perc Endo Approach | Table 3 | Aneurysm coiling |  |
| ICD10 | 03VL0ZZ | 03VL0ZZ - Restriction of Left Internal Carotid Artery, Open Approach | Table 3 | Aneurysm coiling |  |
| ICD10 | 03VL3ZZ | 03VL3ZZ - Restriction of Left Internal Carotid Artery, Perc Approach | Table 3 | Aneurysm coiling |  |
| ICD10 | 03VL4ZZ | 03VL4ZZ - Restriction of L Int Carotid, Perc Endo Approach | Table 3 | Aneurysm coiling |  |
| ICD10 | 03VP0ZZ | 03VP0ZZ - Restriction of Right Vertebral Artery, Open Approach | Table 3 | Aneurysm coiling |  |
| ICD10 | 03VP3ZZ | 03VP3ZZ - Restriction of Right Vertebral Artery, Percutaneous Approach | Table 3 | Aneurysm coiling |  |
| ICD10 | 03VP4ZZ | 03VP4ZZ - Restriction of Right Vertebral Artery, Perc Endo Approach | Table 3 | Aneurysm coiling |  |
| ICD10 | 03VQ0ZZ | 03VQ0ZZ - Restriction of Left Vertebral Artery, Open Approach | Table 3 | Aneurysm coiling |  |
| ICD10 | 03VQ3ZZ | 03VQ3ZZ - Restriction of Left Vertebral Artery, Percutaneous Approach | Table 3 | Aneurysm coiling |  |
| ICD10 | 03VQ4ZZ | 03VQ4ZZ - Restriction of Left Vertebral Artery, Perc Endo Approach | Table 3 | Aneurysm coiling |  |
| ICD9 | 0062 | 0062 - PERC ANGIO IC VESSEL | Table 3 | Angio-plasty (Cerebral) |  |
| ICD10 | 037G34Z | 037G34Z - Dilate of Intracran Art with Drug-elut Intra, Perc Approach | Table 3 | Angio-plasty (Cerebral) |  |
| ICD10 | 037G3DZ | 037G3DZ - Dilation of Intracran Art with Intralum Dev, Perc Approach | Table 3 | Angio-plasty (Cerebral) |  |
| ICD10 | 037G3ZZ | 037G3ZZ - Dilation of Intracranial Artery, Percutaneous Approach | Table 3 | Angio-plasty (Cerebral) |  |
| ICD10 | 037G44Z | 037G44Z - Dilate Intracran Art w Drug-elut Intra, Perc Endo | Table 3 | Angio-plasty (Cerebral) |  |
| ICD10 | 037G4DZ | 037G4DZ - Dilate Intracran Art w Intralum Dev, Perc Endo | Table 3 | Angio-plasty (Cerebral) |  |
| ICD10 | 037G4ZZ | 037G4ZZ - Dilation of Intracranial Artery, Perc Endo Approach | Table 3 | Angio-plasty (Cerebral) |  |
| ICD10 | 03CG3ZZ | 03CG3ZZ - Extirpation of Matter from Intracran Art, Perc Approach | Table 3 | Angio-plasty (Cerebral) |  |
| ICD10 | 057L3DZ | 057L3DZ - Dilation of Intracran Vein with Intralum Dev, Perc Approach | Table 3 | Angio-plasty (Cerebral) |  |
| ICD10 | 057L4DZ | 057L4DZ - Dilate Intracran Vein w Intralum Dev, Perc Endo | Table 3 | Angio-plasty (Cerebral) |  |
| ICD10 | 05CL3ZZ | 05CL3ZZ - Extirpation of Matter from Intracranial Vein, Perc Approach | Table 3 | Angio-plasty (Cerebral) |  |
| ICD9 | 51882 | 51882 - OTHER PULMONARY INSUFF | Table 3 | ARDS |  |
| ICD10 | J80 | J80 - Acute respiratory distress syndrome | Table 3 | ARDS |  |
| ICD10 | N17 | N17 - Acute kidney failure | Table 3 | ARF/AKI |  |
| ICD10 | N170 | N170 - Acute kidney failure with tubular necrosis | Table 3 | ARF/AKI |  |
| ICD10 | N171 | N171 - Acute kidney failure with acute cortical necrosis | Table 3 | ARF/AKI |  |
| ICD10 | N172 | N172 - Acute kidney failure with medullary necrosis | Table 3 | ARF/AKI |  |
| ICD10 | N178 | N178 - Other acute kidney failure | Table 3 | ARF/AKI |  |
| ICD10 | N179 | N179 - Acute kidney failure, unspecified | Table 3 | ARF/AKI |  |
| ICD9 | 5836 | 5836 - RENAL CORT NECROSIS NOS | Table 3 | ARF/AKI |  |
| ICD9 | 5837 | 5837 - NEPHR NOS/MEDULL NECROS | Table 3 | ARF/AKI |  |
| ICD9 | 5845 | 5845 - AC KF W TUBULAR NEPHR | Table 3 | ARF/AKI |  |
| ICD9 | 5846 | 5846 - AC KF W CORT NECROSIS | Table 3 | ARF/AKI |  |
| ICD9 | 5847 | 5847 - AC KF W MEDULL NECROSIS | Table 3 | ARF/AKI |  |
| ICD9 | 5848 | 5848 - ACUTE KIDNEY FAILURE NEC | Table 3 | ARF/AKI |  |
| ICD9 | 5849 | 5849 - ACUTE KIDNEY FAILURE NOS | Table 3 | ARF/AKI |  |
| ICD9 | 8841 | 8841 - CEREBRAL ARTERIOGRAM | Table 3 | Cerebral Angiogram |  |
| ICD10 | B3060ZZ | B3060ZZ - Plain Radiography of R Int Carotid using H Osm Contrast | Table 3 | Cerebral Angiogram |  |
| ICD10 | B3061ZZ | B3061ZZ - Plain Radiography of R Int Carotid using L Osm Contrast | Table 3 | Cerebral Angiogram |  |
| ICD10 | B306YZZ | B306YZZ - Plain Radiography of R Int Carotid using Oth Contrast | Table 3 | Cerebral Angiogram |  |
| ICD10 | B3070ZZ | B3070ZZ - Plain Radiography of L Int Carotid using H Osm Contrast | Table 3 | Cerebral Angiogram |  |
| ICD10 | B3071ZZ | B3071ZZ - Plain Radiography of L Int Carotid using L Osm Contrast | Table 3 | Cerebral Angiogram |  |
| ICD10 | B307YZZ | B307YZZ - Plain Radiography of L Int Carotid using Oth Contrast | Table 3 | Cerebral Angiogram |  |
| ICD10 | B3080ZZ | B3080ZZ - Plain Radiography of Bi Int Carotid using H Osm Contrast | Table 3 | Cerebral Angiogram |  |
| ICD10 | B3081ZZ | B3081ZZ - Plain Radiography of Bi Int Carotid using L Osm Contrast | Table 3 | Cerebral Angiogram |  |
| ICD10 | B308YZZ | B308YZZ - Plain Radiography of Bi Int Carotid using Oth Contrast | Table 3 | Cerebral Angiogram |  |
| ICD10 | B30D0ZZ | B30D0ZZ - Plain Radiography of R Verteb Art using H Osm Contrast | Table 3 | Cerebral Angiogram |  |
| ICD10 | B30D1ZZ | B30D1ZZ - Plain Radiography of R Verteb Art using L Osm Contrast | Table 3 | Cerebral Angiogram |  |
| ICD10 | B30DYZZ | B30DYZZ - Plain Radiography of R Verteb Art using Oth Contrast | Table 3 | Cerebral Angiogram |  |
| ICD10 | B30F0ZZ | B30F0ZZ - Plain Radiography of L Verteb Art using H Osm Contrast | Table 3 | Cerebral Angiogram |  |
| ICD10 | B30F1ZZ | B30F1ZZ - Plain Radiography of L Verteb Art using L Osm Contrast | Table 3 | Cerebral Angiogram |  |
| ICD10 | B30FYZZ | B30FYZZ - Plain Radiography of L Verteb Art using Oth Contrast | Table 3 | Cerebral Angiogram |  |
| ICD10 | B30G0ZZ | B30G0ZZ - Plain Radiography of Bi Verteb Art using H Osm Contrast | Table 3 | Cerebral Angiogram |  |
| ICD10 | B30G1ZZ | B30G1ZZ - Plain Radiography of Bi Verteb Art using L Osm Contrast | Table 3 | Cerebral Angiogram |  |
| ICD10 | B30GYZZ | B30GYZZ - Plain Radiography of Bi Verteb Art using Oth Contrast | Table 3 | Cerebral Angiogram |  |
| ICD10 | B30R0ZZ | B30R0ZZ - Plain Radiography of Intracran Art using H Osm Contrast | Table 3 | Cerebral Angiogram |  |
| ICD10 | B30R1ZZ | B30R1ZZ - Plain Radiography of Intracran Art using L Osm Contrast | Table 3 | Cerebral Angiogram |  |
| ICD10 | B30RYZZ | B30RYZZ - Plain Radiography of Intracran Art using Oth Contrast | Table 3 | Cerebral Angiogram |  |
| ICD10 | B30RZZZ | B30RZZZ - Plain Radiography of Intracranial Arteries | Table 3 | Cerebral Angiogram |  |
| ICD10 | B3160ZZ | B3160ZZ - Fluoroscopy of R Int Carotid using H Osm Contrast | Table 3 | Cerebral Angiogram |  |
| ICD10 | B3161ZZ | B3161ZZ - Fluoroscopy of R Int Carotid using L Osm Contrast | Table 3 | Cerebral Angiogram |  |
| ICD10 | B316YZZ | B316YZZ - Fluoroscopy of R Int Carotid using Oth Contrast | Table 3 | Cerebral Angiogram |  |
| ICD10 | B3170ZZ | B3170ZZ - Fluoroscopy of L Int Carotid using H Osm Contrast | Table 3 | Cerebral Angiogram |  |
| ICD10 | B3171ZZ | B3171ZZ - Fluoroscopy of L Int Carotid using L Osm Contrast | Table 3 | Cerebral Angiogram |  |
| ICD10 | B317YZZ | B317YZZ - Fluoroscopy of L Int Carotid using Oth Contrast | Table 3 | Cerebral Angiogram |  |
| ICD10 | B3180ZZ | B3180ZZ - Fluoroscopy of Bi Int Carotid using H Osm Contrast | Table 3 | Cerebral Angiogram |  |
| ICD10 | B3181ZZ | B3181ZZ - Fluoroscopy of Bi Int Carotid using L Osm Contrast | Table 3 | Cerebral Angiogram |  |
| ICD10 | B318YZZ | B318YZZ - Fluoroscopy of Bi Int Carotid using Oth Contrast | Table 3 | Cerebral Angiogram |  |
| ICD10 | B31D0ZZ | B31D0ZZ - Fluoroscopy of Right Vertebral Artery using H Osm Contrast | Table 3 | Cerebral Angiogram |  |
| ICD10 | B31D1ZZ | B31D1ZZ - Fluoroscopy of Right Vertebral Artery using L Osm Contrast | Table 3 | Cerebral Angiogram |  |
| ICD10 | B31DYZZ | B31DYZZ - Fluoroscopy of Right Vertebral Artery using Other Contrast | Table 3 | Cerebral Angiogram |  |
| ICD10 | B31F0ZZ | B31F0ZZ - Fluoroscopy of Left Vertebral Artery using H Osm Contrast | Table 3 | Cerebral Angiogram |  |
| ICD10 | B31F1ZZ | B31F1ZZ - Fluoroscopy of Left Vertebral Artery using L Osm Contrast | Table 3 | Cerebral Angiogram |  |
| ICD10 | B31FYZZ | B31FYZZ - Fluoroscopy of Left Vertebral Artery using Other Contrast | Table 3 | Cerebral Angiogram |  |
| ICD10 | B31G0ZZ | B31G0ZZ - Fluoroscopy of Bi Verteb Art using H Osm Contrast | Table 3 | Cerebral Angiogram |  |
| ICD10 | B31G1ZZ | B31G1ZZ - Fluoroscopy of Bi Verteb Art using L Osm Contrast | Table 3 | Cerebral Angiogram |  |
| ICD10 | B31GYZZ | B31GYZZ - Fluoroscopy of Bi Verteb Art using Oth Contrast | Table 3 | Cerebral Angiogram |  |
| ICD10 | B31R0ZZ | B31R0ZZ - Fluoroscopy of Intracranial Arteries using H Osm Contrast | Table 3 | Cerebral Angiogram |  |
| ICD10 | B31R1ZZ | B31R1ZZ - Fluoroscopy of Intracranial Arteries using L Osm Contrast | Table 3 | Cerebral Angiogram |  |
| ICD10 | B31RYZZ | B31RYZZ - Fluoroscopy of Intracranial Arteries using Other Contrast | Table 3 | Cerebral Angiogram |  |
| ICD10 | B31RZZZ | B31RZZZ - Fluoroscopy of Intracranial Arteries | Table 3 | Cerebral Angiogram |  |
| ICD10 |  | Cerebral Angiogram | Table 3 | Cerebral Angiogram |  |
| ICD9 | 3485 | 3485 - CEREBRAL EDEMA | Table 3 | Cerebral Edema |  |
| ICD10 | G936 | G936 - Cerebral edema | Table 3 | Cerebral Edema |  |
| ICD10 | S061X0A | S061X0A - Traumatic cerebral edema w/o loss of consciousness, init | Table 3 | Cerebral Edema |  |
| ICD10 | S061X1A | S061X1A - Traumatic cerebral edema w LOC of 30 minutes or less, init | Table 3 | Cerebral Edema |  |
| ICD10 | S061X2A | S061X2A - Traumatic cerebral edema w LOC of 31-59 min, init | Table 3 | Cerebral Edema |  |
| ICD10 | S061X3A | S061X3A - Traumatic cerebral edema w LOC of 1-5 hrs 59 min, init | Table 3 | Cerebral Edema |  |
| ICD10 | S061X4A | S061X4A - Traumatic cerebral edema w LOC of 6 hours to 24 hours, init | Table 3 | Cerebral Edema |  |
| ICD10 | S061X5A | S061X5A - Traumatic cerebral edema w LOC >24 hr w ret consc lev, init | Table 3 | Cerebral Edema |  |
| ICD10 | S061X6A | S061X6A - Traum cerebral edema w LOC >24 hr w/o ret consc w surv, init | Table 3 | Cerebral Edema |  |
| ICD10 | S061X7A | S061X7A - Traum cereb edema w LOC w death d/t brain inj bf consc, init | Table 3 | Cerebral Edema |  |
| ICD10 | S061X8A | S061X8A - Traum cereb edema w LOC w death d/t oth cause bf consc, init | Table 3 | Cerebral Edema |  |
| ICD10 | S061X9A | S061X9A - Traumatic cerebral edema w LOC of unsp duration, init | Table 3 | Cerebral Edema |  |
| ICD9 | 4280 | 4280 - CHF NOS | Table 3 | CHF |  |
| ICD9 | 42820 | 42820 - SYSTOLIC HF NOS | Table 3 | CHF |  |
| ICD9 | 42821 | 42821 - ACUTE SYSTOLIC HF | Table 3 | CHF |  |
| ICD9 | 42822 | 42822 - CHRONIC SYSTOLIC HF | Table 3 | CHF |  |
| ICD9 | 42823 | 42823 - AC & CHR SYSTOLIC HF | Table 3 | CHF |  |
| ICD9 | 42830 | 42830 - DIASTOLIC HF NOS | Table 3 | CHF |  |
| ICD9 | 42831 | 42831 - ACUTE DIASTOLIC HF | Table 3 | CHF |  |
| ICD9 | 42832 | 42832 - CHRONIC DIASTOLIC HF | Table 3 | CHF |  |
| ICD9 | 42833 | 42833 - AC & CHR DIASTOLIC HF | Table 3 | CHF |  |
| ICD9 | 42840 | 42840 - SYS & DIASTOLIC HF NOS | Table 3 | CHF |  |
| ICD9 | 42841 | 42841 - AC SYS & DIASTOLIC HF | Table 3 | CHF |  |
| ICD9 | 42842 | 42842 - CHR SYS & DIASTOLIC HF | Table 3 | CHF |  |
| ICD9 | 42843 | 42843 - ACCHR SYS & DIASTOLIC HF | Table 3 | CHF |  |
| ICD10 | I5020 | I5020 - Unspecified systolic (congestive) heart failure | Table 3 | CHF |  |
| ICD10 | I5021 | I5021 - Acute systolic (congestive) heart failure | Table 3 | CHF |  |
| ICD10 | I5022 | I5022 - Chronic systolic (congestive) heart failure | Table 3 | CHF |  |
| ICD10 | I5023 | I5023 - Acute on chronic systolic (congestive) heart failure | Table 3 | CHF |  |
| ICD10 | I5030 | I5030 - Unspecified diastolic (congestive) heart failure | Table 3 | CHF |  |
| ICD10 | I5031 | I5031 - Acute diastolic (congestive) heart failure | Table 3 | CHF |  |
| ICD10 | I5032 | I5032 - Chronic diastolic (congestive) heart failure | Table 3 | CHF |  |
| ICD10 | I5033 | I5033 - Acute on chronic diastolic (congestive) heart failure | Table 3 | CHF |  |
| ICD10 | I5040 | I5040 - Unsp combined systolic and diastolic (congestive) hrt fail | Table 3 | CHF |  |
| ICD10 | I5041 | I5041 - Acute combined systolic and diastolic (congestive) hrt fail | Table 3 | CHF |  |
| ICD10 | I5042 | I5042 - Chronic combined systolic and diastolic hrt fail | Table 3 | CHF |  |
| ICD10 | I5043 | I5043 - Acute on chronic combined systolic and diastolic hrt fail | Table 3 | CHF |  |
| Complication | 2 | Aspiration Pneumonia | Table 3 | Complications |  |
| Complication | 32 | Aspiration Pneumonia | Table 3 | Complications |  |
| Complication | 62 | Aspiration Pneumonia | Table 3 | Complications |  |
| Complication | 69 | C.diff enteritis (hospital acquired) | Table 3 | Complications |  |
| VizientCode | 95827 | Continuous EEG monitoring | Table 3 | Continuous EEG monitoring |  |
| VizientCode | 70450 | ct head/brain w/o contrast | Table 3 | CT scans | CRM Minor 4102 |
| VizientCode | 70460 | ct head/brain w/contrast | Table 3 | CT scans | CRM Minor 4102 |
| VizientCode | 70470 | ct head/brain w/o & w/contrast | Table 3 | CT scans | CRM Minor 4102 |
| VizientCode | 70496 | ct angiography head | Table 3 | CTA | CRM Minor 4101 |
| VizientCode | 70498 | ct angiography neck | Table 3 | CTA | CRM Minor 4101 |
|  |  |  | Table 3 | CTP |  |
| ICD10 | 4A04XB1 | 4A04XB1 - Measurement of Venous Pressure, Peripheral, Extern Approach | Table 3 | CVP monitoring |  |
| ICD10 | 4A14XB1 | 4A14XB1 - Monitoring of Venous Pressure, Peripheral, External Approach | Table 3 | CVP monitoring |  |
| ICD9 | 8962 | 8962 - CVP MONITORING | Table 3 | CVP monitoring |  |
| ICD9 | 45350 | 45350 - CHR DVT LEG NOS | Table 3 | DVT |  |
| ICD10 | I82401 | I82401 - Acute embolism and thombos unsp deep veins of r low extrem | Table 3 | DVT |  |
| ICD10 | I82402 | I82402 - Acute embolism and thombos unsp deep veins of l low extrem | Table 3 | DVT |  |
| ICD10 | I82403 | I82403 - Acute embolism and thombos unsp deep veins of low extrm, bi | Table 3 | DVT |  |
| ICD10 | I82409 | I82409 - Acute embolism and thombos unsp deep vn unsp lower extremity | Table 3 | DVT |  |
| VizientCode | 95812 | eeg 41-60 minutes | Table 3 | EEG | CRM Minor 8901 |
| VizientCode | 95813 | eeg over 1 hour | Table 3 | EEG | CRM Minor 8901 |
| VizientCode | 95816 | eeg awake and drowsy | Table 3 | EEG | CRM Minor 8901 |
| VizientCode | 95819 | eeg awake and asleep | Table 3 | EEG | CRM Minor 8901 |
| VizientCode | 95822 | eeg coma or sleep only | Table 3 | EEG | CRM Minor 8901 |
| VizientCode | 95824 | eeg cerebral death only | Table 3 | EEG | CRM Minor 8901 |
| VizientCode | 95827 | eeg all night recording | Table 3 | EEG | CRM Minor 8901 |
| ICD9 | 0221 | Insertion or replacement of external ventricular drain [EVD] | Table 2 | EVD |  |
| ICD10 | [009600Z](http://www.icd10data.com/ICD10PCS/Codes/0/0/9/6/009600Z) | Drainage of Cerebral Ventricle with Drainage Device, Open Approach | Table 2 | EVD |  |
| ICD10 | [009630Z](http://www.icd10data.com/ICD10PCS/Codes/0/0/9/6/009630Z) | Drainage of Cerebral Ventricle with Drainage Device, Percutaneous Approach | Table 2 | EVD |  |
| ICD10 | [009640Z](http://www.icd10data.com/ICD10PCS/Codes/0/0/9/6/009640Z) | Drainage of Cerebral Ventricle with Drainage Device, Percutaneous Endoscopic Approach | Table 2 | EVD |  |
| ICD9 | 3313 | 3313 - COMMUNIC HYDROCEPHALUS | Table 3 | Hydro-cephalus |  |
| ICD9 | 3314 | 3314 - OBSTR HYDROCEPHALUS | Table 3 | Hydro-cephalus |  |
| ICD10 | G910 | G910 - Communicating hydrocephalus | Table 3 | Hydro-cephalus |  |
| ICD10 | G911 | G911 - Obstructive hydrocephalus | Table 3 | Hydro-cephalus |  |
| ICD10 | G913 | G913 - Post-traumatic hydrocephalus, unspecified | Table 3 | Hydro-cephalus |  |
| ICD10 | G914 | G914 - Hydrocephalus in diseases classified elsewhere | Table 3 | Hydro-cephalus |  |
| ICD10 | G918 | G918 - Other hydrocephalus | Table 3 | Hydro-cephalus |  |
| ICD10 | G919 | G919 - Hydrocephalus, unspecified | Table 3 | Hydro-cephalus |  |
| ICD9 | 2761 | 2761 - HYPOSMOLALITY | Table 3 | Hypo-natremia |  |
| ICD10 | E871 | E871 - Hypo-osmolality and hyponatremia | Table 3 | Hypo-natremia |  |
| ICD9 | 0110 | 0110 - IC PRESSURE MONITORING | Table 3 | ICP monitor (other than EVD)** |  |
| ICD10 | 4A003BD | 4A003BD - Measurement of Intracranial Pressure, Percutaneous Approach | Table 3 | ICP monitor (other than EVD)** |  |
| ICD10 | 4A007BD | 4A007BD - Measurement of Intracranial Pressure, Via Opening | Table 3 | ICP monitor (other than EVD)** |  |
| ICD10 | 4A103BD | 4A103BD - Monitoring of Intracranial Pressure, Percutaneous Approach | Table 3 | ICP monitor (other than EVD)** |  |
| ICD10 | 4A107BD | 4A107BD - Monitoring of Intracranial Pressure, Via Opening | Table 3 | ICP monitor (other than EVD)** |  |
| Complication | 38 | Acute MI during stay | Table 3 | MI (STEMI/ NSTEMI) |  |
| Complication | 38 | Acute MI during stay | Table 3 | MI (STEMI/ NSTEMI) |  |
| ICD10 | 4A12X9Z | Monitoring of Cardiac Output, External Approach | Table 3 | Monitoring |  |
| ICD9 | 8967 | Monitoring of cardiac output by oxygen consumption technique | Table 3 | Monitoring |  |
| ICD9 | 8968 | Monitoring of cardiac output by other technique | Table 3 | Monitoring |  |
| VizientCode | 819025 | PA Catheter (Swan Ganz) | Table 3 | Monitoring |  |
| VizientCode | 70551 | mri brain, including brain stem w/o contrast | Table 3 | MRI/MRA | CRM Minor 4105 |
| VizientCode | 70552 | mri brain, including brain stem w contrast | Table 3 | MRI/MRA | CRM Minor 4105 |
| VizientCode | 70553 | mri brain, including brain stem w/o & w contrast | Table 3 | MRI/MRA | CRM Minor 4105 |
| VizientCode | 70554 | mri functional mri brain by tech | Table 3 | MRI/MRA | CRM Minor 4105 |
| VizientCode | 70555 | mri functional mri brain by physician/psychologist | Table 3 | MRI/MRA | CRM Minor 4105 |
| VizientCode | 70557 | mri brain (open intracranial op) w/o contarst | Table 3 | MRI/MRA | CRM Minor 4105 |
| VizientCode | 70558 | mri brain (open intracranial op) w contrast | Table 3 | MRI/MRA | CRM Minor 4105 |
| VizientCode | 70559 | mri brain (open intracranial op) w/o & w contr | Table 3 | MRI/MRA | CRM Minor 4105 |
| ICD9 | 4150 | 4150 - ACUTE COR PULMONALE | Table 3 | PE |  |
| ICD9 | 41512 | 41512 - SEPTIC PULMON EMBOLISM | Table 3 | PE |  |
| ICD9 | 41513 | 41513 - PA SADDLE EMBOLUS | Table 3 | PE |  |
| ICD9 | 41519 | 41519 - PULMON EMBOL/INFARCT NEC | Table 3 | PE |  |
| ICD10 | I2601 | I2601 - Septic pulmonary embolism with acute cor pulmonale | Table 3 | PE |  |
| ICD10 | I2602 | I2602 - Saddle embolus of pulmonary artery with acute cor pulmonale | Table 3 | PE |  |
| ICD10 | I2609 | I2609 - Other pulmonary embolism with acute cor pulmonale | Table 3 | PE |  |
| ICD10 | I2690 | I2690 - Septic pulmonary embolism without acute cor pulmonale | Table 3 | PE |  |
| ICD10 | I2692 | I2692 - Saddle embolus of pulmonary artery w/o acute cor pulmonale | Table 3 | PE |  |
| ICD10 | I2699 | I2699 - Other pulmonary embolism without acute cor pulmonale | Table 3 | PE |  |
| ICD9 | V441 | V441 - GASTROSTOMY STATUS | Table 3 | PEG tube |  |
| ICD10 | Z931 | Z931 - Gastrostomy status | Table 3 | PEG tube |  |
| VizientCode | 013220 | Albumin | Table 3 | Pharmacy |  |
| VizientCode | 185600 | Dobu-tamine | Table 3 | Pharmacy |  |
| VizientCode | 188945 | Dopamine | Table 3 | Pharmacy |  |
| VizientCode | 209720 | Epi-nephrine | Table 3 | Pharmacy |  |
| VizientCode | 376000 | IA Milnirone | Table 3 | Pharmacy |  |
| VizientCode | 404550 | IA Nicardipine | Table 3 | Pharmacy |  |
| VizientCode | 406500 | IA Nimodipine | Table 3 | Pharmacy |  |
| VizientCode | 441770 | IA Papaverine | Table 3 | Pharmacy |  |
| VizientCode | 653400 | IA Verapmil | Table 3 | Pharmacy |  |
| VizientCode | 924782 | Laco-samide | Table 3 | Pharmacy |  |
| VizientCode | 702531 | Leve-tiracetam | Table 3 | Pharmacy |  |
| VizientCode | 376000 | Milnirone | Table 3 | Pharmacy |  |
| VizientCode | 406500 | Nimodipine | Table 3 | Pharmacy |  |
| VizientCode | 410190 | Norepi-nephrine | Table 3 | Pharmacy |  |
| VizientCode | 464840 | Phenyl-ephrine | Table 3 | Pharmacy |  |
| VizientCode | 465045 | Phenytoin | Table 3 | Pharmacy |  |
| VizientCode | 923833 | Fos-phenytoin | Table 3 | Pharmacy |  |
| VizientCode | 924272 | Sodium Valproate | Table 3 | Pharmacy |  |
| VizientCode | 648667 | Valproic acid | Table 3 | Pharmacy |  |
| ICD9 | 5184 | 5184 - ACUTE LUNG EDEMA NOS | Table 3 | Pulmonary Edema |  |
| ICD10 | J810 | J810 - Acute pulmonary edema | Table 3 | Pulmonary Edema |  |
| ICD9 | 51851 | 51851 - AC RESP FAIL TRAUM/SURG | Table 3 | Respiratory Failure |  |
| ICD9 | 51853 | 51853 - A&C RESP FAIL TRAUM/SURG | Table 3 | Respiratory Failure |  |
| ICD9 | 51881 | 51881 - AC RESPIRATORY FAILURE | Table 3 | Respiratory Failure |  |
| ICD9 | 51883 | 51883 - CHR RESPIRATORY FAILURE | Table 3 | Respiratory Failure |  |
| ICD9 | 51884 | 51884 - AC & CHR RESP FAILURE | Table 3 | Respiratory Failure |  |
| ICD10 | J9600 | J9600 - Acute respiratory failure, unsp w hypoxia or hypercapnia | Table 3 | Respiratory Failure |  |
| ICD10 | J9601 | J9601 - Acute respiratory failure with hypoxia | Table 3 | Respiratory Failure |  |
| ICD10 | J9602 | J9602 - Acute respiratory failure with hypercapnia | Table 3 | Respiratory Failure |  |
| ICD10 | J9610 | J9610 - Chronic respiratory failure, unsp w hypoxia or hypercapnia | Table 3 | Respiratory Failure |  |
| ICD10 | J9611 | J9611 - Chronic respiratory failure with hypoxia | Table 3 | Respiratory Failure |  |
| ICD10 | J9612 | J9612 - Chronic respiratory failure with hypercapnia | Table 3 | Respiratory Failure |  |
| ICD10 | J9620 | J9620 - Acute and chr resp failure, unsp w hypoxia or hypercapnia | Table 3 | Respiratory Failure |  |
| ICD10 | J9621 | J9621 - Acute and chronic respiratory failure with hypoxia | Table 3 | Respiratory Failure |  |
| ICD10 | J9622 | J9622 - Acute and chronic respiratory failure with hypercapnia | Table 3 | Respiratory Failure |  |
| ICD10 | J9690 | J9690 - Respiratory failure, unsp, unsp w hypoxia or hypercapnia | Table 3 | Respiratory Failure |  |
| ICD10 | J9691 | J9691 - Respiratory failure, unspecified with hypoxia | Table 3 | Respiratory Failure |  |
| ICD10 | J9692 | J9692 - Respiratory failure, unspecified with hypercapnia | Table 3 | Respiratory Failure |  |
| ICD9 | 34500 | 34500 - PETIT MAL W/O INTRACT | Table 3 | Seizures/SE |  |
| ICD9 | 34501 | 34501 - PETIT MAL W INTRACT EPIL | Table 3 | Seizures/SE |  |
| ICD9 | 34510 | 34510 - GRAND MAL W/O INTRACT | Table 3 | Seizures/SE |  |
| ICD9 | 34511 | 34511 - GRAND MAL W INTRACT EPIL | Table 3 | Seizures/SE |  |
| ICD9 | 3452 | 3452 - PETIT MAL STATUS | Table 3 | Seizures/SE |  |
| ICD9 | 3453 | 3453 - GRAND MAL STATUS | Table 3 | Seizures/SE |  |
| ICD9 | 34540 | 34540 - LRE W CPS W/O INTRACT | Table 3 | Seizures/SE |  |
| ICD9 | 34541 | 34541 - LRE W CPS W INTRACT | Table 3 | Seizures/SE |  |
| ICD9 | 34550 | 34550 - LRE W SPS W/O INTRACT | Table 3 | Seizures/SE |  |
| ICD9 | 34551 | 34551 - LRE W SPS W INTRACT | Table 3 | Seizures/SE |  |
| ICD9 | 34570 | 34570 - EPIL PART CONT S INTRACT | Table 3 | Seizures/SE |  |
| ICD9 | 34571 | 34571 - INTRACT EPIL PART CONT | Table 3 | Seizures/SE |  |
| ICD9 | 34580 | 34580 - EPILEPSY NEC W/O INTRACT | Table 3 | Seizures/SE |  |
| ICD9 | 34581 | 34581 - INTRACTABLE EPILEPSY NEC | Table 3 | Seizures/SE |  |
| ICD9 | 34590 | 34590 - EPILEPSY NOS W/O INTRACT | Table 3 | Seizures/SE |  |
| ICD9 | 34591 | 34591 - INTRACTABLE EPILEPSY NOS | Table 3 | Seizures/SE |  |
| ICD9 | 78039 | 78039 - OTHER CONVULSIONS | Table 3 | Seizures/SE |  |
| ICD10 | G40001 | G40001 - Local-rel idio epi w seiz of loc onst, not ntrct, w stat epi | Table 3 | Seizures/SE |  |
| ICD10 | G40009 | G40009 - Local-rel idio epi w seiz of loc onst,not ntrct,w/o stat epi | Table 3 | Seizures/SE |  |
| ICD10 | G40011 | G40011 - Local-rel idio epi w seiz of loc onset, ntrct, w stat epi | Table 3 | Seizures/SE |  |
| ICD10 | G40019 | G40019 - Local-rel idio epi w seiz of loc onset, ntrct, w/o stat epi | Table 3 | Seizures/SE |  |
| ICD10 | G40101 | G40101 - Local-rel symptc epi w simp part seiz, not ntrct, w stat epi | Table 3 | Seizures/SE |  |
| ICD10 | G40109 | G40109 - Local-rel symptc epi w simp prt seiz,not ntrct, w/o stat epi | Table 3 | Seizures/SE |  |
| ICD10 | G40111 | G40111 - Local-rel symptc epi w simple part seiz, ntrct, w stat epi | Table 3 | Seizures/SE |  |
| ICD10 | G40119 | G40119 - Local-rel symptc epi w simple part seiz, ntrct, w/o stat epi | Table 3 | Seizures/SE |  |
| ICD10 | G40201 | G40201 - Local-rel symptc epi w cmplx prt seiz, not ntrct, w stat epi | Table 3 | Seizures/SE |  |
| ICD10 | G40209 | G40209 - Local-rel symptc epi w cmplx prt seiz,not ntrct,w/o stat epi | Table 3 | Seizures/SE |  |
| ICD10 | G40211 | G40211 - Local-rel symptc epi w cmplx partial seiz, ntrct, w stat epi | Table 3 | Seizures/SE |  |
| ICD10 | G40219 | G40219 - Local-rel symptc epi w cmplx part seiz, ntrct, w/o stat epi | Table 3 | Seizures/SE |  |
| ICD10 | G40301 | G40301 - Gen idiopathic epilepsy, not intractable, w stat epi | Table 3 | Seizures/SE |  |
| ICD10 | G40309 | G40309 - Gen idiopathic epilepsy, not intractable, w/o stat epi | Table 3 | Seizures/SE |  |
| ICD10 | G40311 | G40311 - Generalized idiopathic epilepsy, intractable, w stat epi | Table 3 | Seizures/SE |  |
| ICD10 | G40319 | G40319 - Generalized idiopathic epilepsy, intractable, w/o stat epi | Table 3 | Seizures/SE |  |
| ICD10 | G40401 | G40401 - Oth generalized epilepsy, not intractable, w stat epi | Table 3 | Seizures/SE |  |
| ICD10 | G40409 | G40409 - Oth generalized epilepsy, not intractable, w/o stat epi | Table 3 | Seizures/SE |  |
| ICD10 | G40411 | G40411 - Oth generalized epilepsy, intractable, w status epilepticus | Table 3 | Seizures/SE |  |
| ICD10 | G40419 | G40419 - Oth generalized epilepsy, intractable, w/o stat epi | Table 3 | Seizures/SE |  |
| ICD10 | G40501 | G40501 - Epileptic seiz rel to extrn causes, not ntrct, w stat epi | Table 3 | Seizures/SE |  |
| ICD10 | G40509 | G40509 - Epileptic seiz rel to extrn causes, not ntrct, w/o stat epi | Table 3 | Seizures/SE |  |
| ICD10 | G40801 | G40801 - Other epilepsy, not intractable, with status epilepticus | Table 3 | Seizures/SE |  |
| ICD10 | G40802 | G40802 - Other epilepsy, not intractable, without status epilepticus | Table 3 | Seizures/SE |  |
| ICD10 | G40803 | G40803 - Other epilepsy, intractable, with status epilepticus | Table 3 | Seizures/SE |  |
| ICD10 | G40804 | G40804 - Other epilepsy, intractable, without status epilepticus | Table 3 | Seizures/SE |  |
| ICD10 | G4089 | G4089 - Other seizures | Table 3 | Seizures/SE |  |
| ICD10 | G40901 | G40901 - Epilepsy, unsp, not intractable, with status epilepticus | Table 3 | Seizures/SE |  |
| ICD10 | G40909 | G40909 - Epilepsy, unsp, not intractable, without status epilepticus | Table 3 | Seizures/SE |  |
| ICD10 | G40911 | G40911 - Epilepsy, unspecified, intractable, with status epilepticus | Table 3 | Seizures/SE |  |
| ICD10 | G40919 | G40919 - Epilepsy, unsp, intractable, without status epilepticus | Table 3 | Seizures/SE |  |
| ICD10 | R569 | R569 - Unspecified convulsions | Table 3 | Seizures/SE |  |
| ICD9 | 42983 | 42983 - TAKOTSUBO SYNDROME | Table 3 | Stress CMP/ Takatsubo CMP |  |
| ICD10 | I5181 | I5181 - Takotsubo syndrome | Table 3 | Stress CMP/ Takatsubo CMP |  |
| ICD9 | 303 | 303 - ALCOHOL DEPENDENCE SYND | Table 1 | Substance use - alcohol |  |
| ICD9 | 3030 | 3030 - AC ALCOHOL INTOXICATION | Table 1 | Substance use - alcohol |  |
| ICD9 | 30300 | 30300 - AC ALCOHOL INTOX-UNSPEC | Table 1 | Substance use - alcohol |  |
| ICD9 | 30301 | 30301 - AC ALCOHOL INTOX-CONT | Table 1 | Substance use - alcohol |  |
| ICD9 | 30302 | 30302 - AC ALCOHOL INTOX-EPIS | Table 1 | Substance use - alcohol |  |
| ICD9 | 30303 | 30303 - AC ALCOHOL INTOX-REMISS | Table 1 | Substance use - alcohol |  |
| ICD9 | 3039 | 3039 - ALCOHOL DEP NEC & NOS | Table 1 | Substance use - alcohol |  |
| ICD9 | 30390 | 30390 - ALC DEP NEC & NOS-UNSPEC | Table 1 | Substance use - alcohol |  |
| ICD9 | 30391 | 30391 - ALC DEP NEC & NOS-CONT | Table 1 | Substance use - alcohol |  |
| ICD9 | 30392 | 30392 - ALC DEP NEC & NOS-EPIS | Table 1 | Substance use - alcohol |  |
| ICD9 | 30393 | 30393 - ALC DEP NEC & NOS-REMISS | Table 1 | Substance use - alcohol |  |
| ICD9 | 3050 | 3050 - ALCOHOL ABUSE | Table 1 | Substance use - alcohol |  |
| ICD9 | 30500 | 30500 - ALCOHOL ABUSE-UNSPEC | Table 1 | Substance use - alcohol |  |
| ICD9 | 30501 | 30501 - ALCOHOL ABUSE-CONTINUOUS | Table 1 | Substance use - alcohol |  |
| ICD9 | 30502 | 30502 - ALCOHOL ABUSE-EPISODIC | Table 1 | Substance use - alcohol |  |
| ICD9 | 30503 | 30503 - ALCOHOL ABUSE-IN REMISS | Table 1 | Substance use - alcohol |  |
| ICD10 | F10 | F10 - Alcohol related disorders | Table 1 | Substance use - alcohol |  |
| ICD10 | F101 | F101 - Alcohol abuse | Table 1 | Substance use - alcohol |  |
| ICD10 | F1010 | F1010 - Alcohol abuse, uncomplicated | Table 1 | Substance use - alcohol |  |
| ICD10 | F1011 | F1011 - Alcohol abuse, in remission | Table 1 | Substance use - alcohol |  |
| ICD10 | F1012 | F1012 - Alcohol abuse with intoxication | Table 1 | Substance use - alcohol |  |
| ICD10 | F10120 | F10120 - Alcohol abuse with intoxication, uncomplicated | Table 1 | Substance use - alcohol |  |
| ICD10 | F10121 | F10121 - Alcohol abuse with intoxication delirium | Table 1 | Substance use - alcohol |  |
| ICD10 | F10129 | F10129 - Alcohol abuse with intoxication, unspecified | Table 1 | Substance use - alcohol |  |
| ICD10 | F1014 | F1014 - Alcohol abuse with alcohol-induced mood disorder | Table 1 | Substance use - alcohol |  |
| ICD10 | F1015 | F1015 - Alcohol abuse with alcohol-induced psychotic disorder | Table 1 | Substance use - alcohol |  |
| ICD10 | F10150 | F10150 - Alcohol abuse w alcoh-induce psychotic disorder w delusions | Table 1 | Substance use - alcohol |  |
| ICD10 | F10151 | F10151 - Alcohol abuse w alcoh-induce psychotic disorder w hallucin | Table 1 | Substance use - alcohol |  |
| ICD10 | F10159 | F10159 - Alcohol abuse with alcohol-induced psychotic disorder, unsp | Table 1 | Substance use - alcohol |  |
| ICD10 | F1018 | F1018 - Alcohol abuse with other alcohol-induced disorders | Table 1 | Substance use - alcohol |  |
| ICD10 | F10180 | F10180 - Alcohol abuse with alcohol-induced anxiety disorder | Table 1 | Substance use - alcohol |  |
| ICD10 | F10181 | F10181 - Alcohol abuse with alcohol-induced sexual dysfunction | Table 1 | Substance use - alcohol |  |
| ICD10 | F10182 | F10182 - Alcohol abuse with alcohol-induced sleep disorder | Table 1 | Substance use - alcohol |  |
| ICD10 | F10188 | F10188 - Alcohol abuse with other alcohol-induced disorder | Table 1 | Substance use - alcohol |  |
| ICD10 | F1019 | F1019 - Alcohol abuse with unspecified alcohol-induced disorder | Table 1 | Substance use - alcohol |  |
| ICD10 | F102 | F102 - Alcohol dependence | Table 1 | Substance use - alcohol |  |
| ICD10 | F1020 | F1020 - Alcohol dependence, uncomplicated | Table 1 | Substance use - alcohol |  |
| ICD10 | F1021 | F1021 - Alcohol dependence, in remission | Table 1 | Substance use - alcohol |  |
| ICD10 | F1022 | F1022 - Alcohol dependence with intoxication | Table 1 | Substance use - alcohol |  |
| ICD10 | F10220 | F10220 - Alcohol dependence with intoxication, uncomplicated | Table 1 | Substance use - alcohol |  |
| ICD10 | F10221 | F10221 - Alcohol dependence with intoxication delirium | Table 1 | Substance use - alcohol |  |
| ICD10 | F10229 | F10229 - Alcohol dependence with intoxication, unspecified | Table 1 | Substance use - alcohol |  |
| ICD10 | F1023 | F1023 - Alcohol dependence with withdrawal | Table 1 | Substance use - alcohol |  |
| ICD10 | F10230 | F10230 - Alcohol dependence with withdrawal, uncomplicated | Table 1 | Substance use - alcohol |  |
| ICD10 | F10231 | F10231 - Alcohol dependence with withdrawal delirium | Table 1 | Substance use - alcohol |  |
| ICD10 | F10232 | F10232 - Alcohol dependence w withdrawal with perceptual disturbance | Table 1 | Substance use - alcohol |  |
| ICD10 | F10239 | F10239 - Alcohol dependence with withdrawal, unspecified | Table 1 | Substance use - alcohol |  |
| ICD10 | F1024 | F1024 - Alcohol dependence with alcohol-induced mood disorder | Table 1 | Substance use - alcohol |  |
| ICD10 | F1025 | F1025 - Alcohol dependence with alcohol-induced psychotic disorder | Table 1 | Substance use - alcohol |  |
| ICD10 | F10250 | F10250 - Alcohol depend w alcoh-induce psychotic disorder w delusions | Table 1 | Substance use - alcohol |  |
| ICD10 | F10251 | F10251 - Alcohol depend w alcoh-induce psychotic disorder w hallucin | Table 1 | Substance use - alcohol |  |
| ICD10 | F10259 | F10259 - Alcohol dependence w alcoh-induce psychotic disorder, unsp | Table 1 | Substance use - alcohol |  |
| ICD10 | F1026 | F1026 - Alcohol depend w alcoh-induce persisting amnestic disorder | Table 1 | Substance use - alcohol |  |
| ICD10 | F1027 | F1027 - Alcohol dependence with alcohol-induced persisting dementia | Table 1 | Substance use - alcohol |  |
| ICD10 | F1028 | F1028 - Alcohol dependence with other alcohol-induced disorders | Table 1 | Substance use - alcohol |  |
| ICD10 | F10280 | F10280 - Alcohol dependence with alcohol-induced anxiety disorder | Table 1 | Substance use - alcohol |  |
| ICD10 | F10281 | F10281 - Alcohol dependence with alcohol-induced sexual dysfunction | Table 1 | Substance use - alcohol |  |
| ICD10 | F10282 | F10282 - Alcohol dependence with alcohol-induced sleep disorder | Table 1 | Substance use - alcohol |  |
| ICD10 | F10288 | F10288 - Alcohol dependence with other alcohol-induced disorder | Table 1 | Substance use - alcohol |  |
| ICD10 | F1029 | F1029 - Alcohol dependence with unspecified alcohol-induced disorder | Table 1 | Substance use - alcohol |  |
| ICD10 | F109 | F109 - Alcohol use, unspecified | Table 1 | Substance use - alcohol |  |
| ICD10 | F1092 | F1092 - Alcohol use, unspecified with intoxication | Table 1 | Substance use - alcohol |  |
| ICD10 | F10920 | F10920 - Alcohol use, unspecified with intoxication, uncomplicated | Table 1 | Substance use - alcohol |  |
| ICD10 | F10921 | F10921 - Alcohol use, unspecified with intoxication delirium | Table 1 | Substance use - alcohol |  |
| ICD10 | F10929 | F10929 - Alcohol use, unspecified with intoxication, unspecified | Table 1 | Substance use - alcohol |  |
| ICD10 | F1094 | F1094 - Alcohol use, unspecified with alcohol-induced mood disorder | Table 1 | Substance use - alcohol |  |
| ICD10 | F1095 | F1095 - Alcohol use, unsp with alcohol-induced psychotic disorder | Table 1 | Substance use - alcohol |  |
| ICD10 | F10950 | F10950 - Alcohol use, unsp w alcoh-induce psych disorder w delusions | Table 1 | Substance use - alcohol |  |
| ICD10 | F10951 | F10951 - Alcohol use, unsp w alcoh-induce psych disorder w hallucin | Table 1 | Substance use - alcohol |  |
| ICD10 | F10959 | F10959 - Alcohol use, unsp w alcohol-induced psychotic disorder, unsp | Table 1 | Substance use - alcohol |  |
| ICD10 | F1096 | F1096 - Alcohol use, unsp w alcoh-induce persist amnestic disorder | Table 1 | Substance use - alcohol |  |
| ICD10 | F1097 | F1097 - Alcohol use, unsp with alcohol-induced persisting dementia | Table 1 | Substance use - alcohol |  |
| ICD10 | F1098 | F1098 - Alcohol use, unsp with other alcohol-induced disorders | Table 1 | Substance use - alcohol |  |
| ICD10 | F10980 | F10980 - Alcohol use, unsp with alcohol-induced anxiety disorder | Table 1 | Substance use - alcohol |  |
| ICD10 | F10981 | F10981 - Alcohol use, unsp with alcohol-induced sexual dysfunction | Table 1 | Substance use - alcohol |  |
| ICD10 | F10982 | F10982 - Alcohol use, unspecified with alcohol-induced sleep disorder | Table 1 | Substance use - alcohol |  |
| ICD10 | F10988 | F10988 - Alcohol use, unspecified with other alcohol-induced disorder | Table 1 | Substance use - alcohol |  |
| ICD10 | F1099 | F1099 - Alcohol use, unsp with unspecified alcohol-induced disorder | Table 1 | Substance use - alcohol |  |
| ICD9 | 3058 | 3058 - ANTIDEPRESSANT ABUSE | Table 1 | Substance use - antidepressants |  |
| ICD9 | 30580 | 30580 - ANTIDEPRESSANT ABUSE-NOS | Table 1 | Substance use - antidepressants |  |
| ICD9 | 30581 | 30581 - ANTIDEPRESS ABUSE-CONT | Table 1 | Substance use - antidepressants |  |
| ICD9 | 30582 | 30582 - ANTIDEPRESS ABUSE-EPIS | Table 1 | Substance use - antidepressants |  |
| ICD9 | 30583 | 30583 - ANTIDEPRESS ABUSE-REMISS | Table 1 | Substance use - antidepressants |  |
| ICD9 | 3043 | 3043 - CANNABIS DEPENDENCE | Table 1 | Substance use - cannabis |  |
| ICD9 | 30430 | 30430 - CANNABIS DEP-UNSPEC | Table 1 | Substance use - cannabis |  |
| ICD9 | 30431 | 30431 - CANNABIS DEP-CONT | Table 1 | Substance use - cannabis |  |
| ICD9 | 30432 | 30432 - CANNABIS DEP-EPISODIC | Table 1 | Substance use - cannabis |  |
| ICD9 | 30433 | 30433 - CANNABIS DEP-REMISS | Table 1 | Substance use - cannabis |  |
| ICD9 | 3052 | 3052 - CANNABIS ABUSE | Table 1 | Substance use - cannabis |  |
| ICD9 | 30520 | 30520 - CANNABIS ABUSE-UNSPEC | Table 1 | Substance use - cannabis |  |
| ICD9 | 30521 | 30521 - CANNABIS ABUSE-CONT | Table 1 | Substance use - cannabis |  |
| ICD9 | 30522 | 30522 - CANNABIS ABUSE-EPISODIC | Table 1 | Substance use - cannabis |  |
| ICD9 | 30523 | 30523 - CANNABIS ABUSE-IN REMISS | Table 1 | Substance use - cannabis |  |
| ICD9 | 3042 | 3042 - COCAINE DEPENDENCE | Table 1 | Substance use - cocaine |  |
| ICD9 | 30420 | 30420 - COCAINE DEP-UNSPEC | Table 1 | Substance use - cocaine |  |
| ICD9 | 30421 | 30421 - COCAINE DEP-CONT | Table 1 | Substance use - cocaine |  |
| ICD9 | 30422 | 30422 - COCAINE DEP-EPISODIC | Table 1 | Substance use - cocaine |  |
| ICD9 | 30423 | 30423 - COCAINE DEP-REMISS | Table 1 | Substance use - cocaine |  |
| ICD9 | 3056 | 3056 - COCAINE ABUSE | Table 1 | Substance use - cocaine |  |
| ICD9 | 30560 | 30560 - COCAINE ABUSE-UNSPEC | Table 1 | Substance use - cocaine |  |
| ICD9 | 30561 | 30561 - COCAINE ABUSE-CONTINUOUS | Table 1 | Substance use - cocaine |  |
| ICD9 | 30562 | 30562 - COCAINE ABUSE-EPISODIC | Table 1 | Substance use - cocaine |  |
| ICD9 | 30563 | 30563 - COCAINE ABUSE-IN REMISS | Table 1 | Substance use - cocaine |  |
| ICD10 | F14 | F14 - Cocaine related disorders | Table 1 | Substance use - cocaine |  |
| ICD10 | F141 | F141 - Cocaine abuse | Table 1 | Substance use - cocaine |  |
| ICD10 | F1410 | F1410 - Cocaine abuse, uncomplicated | Table 1 | Substance use - cocaine |  |
| ICD10 | F1411 | F1411 - Cocaine abuse, in remission | Table 1 | Substance use - cocaine |  |
| ICD10 | F1412 | F1412 - Cocaine abuse with intoxication | Table 1 | Substance use - cocaine |  |
| ICD10 | F14120 | F14120 - Cocaine abuse with intoxication, uncomplicated | Table 1 | Substance use - cocaine |  |
| ICD10 | F14121 | F14121 - Cocaine abuse with intoxication with delirium | Table 1 | Substance use - cocaine |  |
| ICD10 | F14122 | F14122 - Cocaine abuse with intoxication with perceptual disturbance | Table 1 | Substance use - cocaine |  |
| ICD10 | F14129 | F14129 - Cocaine abuse with intoxication, unspecified | Table 1 | Substance use - cocaine |  |
| ICD10 | F1414 | F1414 - Cocaine abuse with cocaine-induced mood disorder | Table 1 | Substance use - cocaine |  |
| ICD10 | F1415 | F1415 - Cocaine abuse with cocaine-induced psychotic disorder | Table 1 | Substance use - cocaine |  |
| ICD10 | F14150 | F14150 - Cocaine abuse w cocaine-induc psychotic disorder w delusions | Table 1 | Substance use - cocaine |  |
| ICD10 | F14151 | F14151 - Cocaine abuse w cocaine-induc psychotic disorder w hallucin | Table 1 | Substance use - cocaine |  |
| ICD10 | F14159 | F14159 - Cocaine abuse with cocaine-induced psychotic disorder, unsp | Table 1 | Substance use - cocaine |  |
| ICD10 | F1418 | F1418 - Cocaine abuse with other cocaine-induced disorder | Table 1 | Substance use - cocaine |  |
| ICD10 | F14180 | F14180 - Cocaine abuse with cocaine-induced anxiety disorder | Table 1 | Substance use - cocaine |  |
| ICD10 | F14181 | F14181 - Cocaine abuse with cocaine-induced sexual dysfunction | Table 1 | Substance use - cocaine |  |
| ICD10 | F14182 | F14182 - Cocaine abuse with cocaine-induced sleep disorder | Table 1 | Substance use - cocaine |  |
| ICD10 | F14188 | F14188 - Cocaine abuse with other cocaine-induced disorder | Table 1 | Substance use - cocaine |  |
| ICD10 | F1419 | F1419 - Cocaine abuse with unspecified cocaine-induced disorder | Table 1 | Substance use - cocaine |  |
| ICD10 | F142 | F142 - Cocaine dependence | Table 1 | Substance use - cocaine |  |
| ICD10 | F1420 | F1420 - Cocaine dependence, uncomplicated | Table 1 | Substance use - cocaine |  |
| ICD10 | F1421 | F1421 - Cocaine dependence, in remission | Table 1 | Substance use - cocaine |  |
| ICD10 | F1422 | F1422 - Cocaine dependence with intoxication | Table 1 | Substance use - cocaine |  |
| ICD10 | F14220 | F14220 - Cocaine dependence with intoxication, uncomplicated | Table 1 | Substance use - cocaine |  |
| ICD10 | F14221 | F14221 - Cocaine dependence with intoxication delirium | Table 1 | Substance use - cocaine |  |
| ICD10 | F14222 | F14222 - Cocaine dependence w intoxication w perceptual disturbance | Table 1 | Substance use - cocaine |  |
| ICD10 | F14229 | F14229 - Cocaine dependence with intoxication, unspecified | Table 1 | Substance use - cocaine |  |
| ICD10 | F1423 | F1423 - Cocaine dependence with withdrawal | Table 1 | Substance use - cocaine |  |
| ICD10 | F1424 | F1424 - Cocaine dependence with cocaine-induced mood disorder | Table 1 | Substance use - cocaine |  |
| ICD10 | F1425 | F1425 - Cocaine dependence with cocaine-induced psychotic disorder | Table 1 | Substance use - cocaine |  |
| ICD10 | F14250 | F14250 - Cocaine depend w cocaine-induc psych disorder w delusions | Table 1 | Substance use - cocaine |  |
| ICD10 | F14251 | F14251 - Cocaine depend w cocaine-induc psychotic disorder w hallucin | Table 1 | Substance use - cocaine |  |
| ICD10 | F14259 | F14259 - Cocaine dependence w cocaine-induc psychotic disorder, unsp | Table 1 | Substance use - cocaine |  |
| ICD10 | F1428 | F1428 - Cocaine dependence with other cocaine-induced disorder | Table 1 | Substance use - cocaine |  |
| ICD10 | F14280 | F14280 - Cocaine dependence with cocaine-induced anxiety disorder | Table 1 | Substance use - cocaine |  |
| ICD10 | F14281 | F14281 - Cocaine dependence with cocaine-induced sexual dysfunction | Table 1 | Substance use - cocaine |  |
| ICD10 | F14282 | F14282 - Cocaine dependence with cocaine-induced sleep disorder | Table 1 | Substance use - cocaine |  |
| ICD10 | F14288 | F14288 - Cocaine dependence with other cocaine-induced disorder | Table 1 | Substance use - cocaine |  |
| ICD10 | F1429 | F1429 - Cocaine dependence with unspecified cocaine-induced disorder | Table 1 | Substance use - cocaine |  |
| ICD10 | F149 | F149 - Cocaine use, unspecified | Table 1 | Substance use - cocaine |  |
| ICD10 | F1490 | F1490 - Cocaine use, unspecified, uncomplicated | Table 1 | Substance use - cocaine |  |
| ICD10 | F1492 | F1492 - Cocaine use, unspecified with intoxication | Table 1 | Substance use - cocaine |  |
| ICD10 | F14920 | F14920 - Cocaine use, unspecified with intoxication, uncomplicated | Table 1 | Substance use - cocaine |  |
| ICD10 | F14921 | F14921 - Cocaine use, unspecified with intoxication delirium | Table 1 | Substance use - cocaine |  |
| ICD10 | F14922 | F14922 - Cocaine use, unsp w intoxication with perceptual disturbance | Table 1 | Substance use - cocaine |  |
| ICD10 | F14929 | F14929 - Cocaine use, unspecified with intoxication, unspecified | Table 1 | Substance use - cocaine |  |
| ICD10 | F1494 | F1494 - Cocaine use, unspecified with cocaine-induced mood disorder | Table 1 | Substance use - cocaine |  |
| ICD10 | F1495 | F1495 - Cocaine use, unsp with cocaine-induced psychotic disorder | Table 1 | Substance use - cocaine |  |
| ICD10 | F14950 | F14950 - Cocaine use, unsp w cocaine-induc psych disorder w delusions | Table 1 | Substance use - cocaine |  |
| ICD10 | F14951 | F14951 - Cocaine use, unsp w cocaine-induc psych disorder w hallucin | Table 1 | Substance use - cocaine |  |
| ICD10 | F14959 | F14959 - Cocaine use, unsp w cocaine-induced psychotic disorder, unsp | Table 1 | Substance use - cocaine |  |
| ICD10 | F1498 | F1498 - Cocaine use, unspecified with oth cocaine-induced disorder | Table 1 | Substance use - cocaine |  |
| ICD10 | F14980 | F14980 - Cocaine use, unsp with cocaine-induced anxiety disorder | Table 1 | Substance use - cocaine |  |
| ICD10 | F14981 | F14981 - Cocaine use, unsp with cocaine-induced sexual dysfunction | Table 1 | Substance use - cocaine |  |
| ICD10 | F14982 | F14982 - Cocaine use, unspecified with cocaine-induced sleep disorder | Table 1 | Substance use - cocaine |  |
| ICD10 | F14988 | F14988 - Cocaine use, unspecified with other cocaine-induced disorder | Table 1 | Substance use - cocaine |  |
| ICD10 | F1499 | F1499 - Cocaine use, unsp with unspecified cocaine-induced disorder | Table 1 | Substance use - cocaine |  |
| ICD9 | 3045 | 3045 - HALLUCINOGEN DEPENDENCE | Table 1 | Substance use - hallucinogen |  |
| ICD9 | 30450 | 30450 - HALLUCINOGEN DEP-UNSPEC | Table 1 | Substance use - hallucinogen |  |
| ICD9 | 30451 | 30451 - HALLUCINOGEN DEP-CONT | Table 1 | Substance use - hallucinogen |  |
| ICD9 | 30452 | 30452 - HALLUCINOGEN DEP-EPIS | Table 1 | Substance use - hallucinogen |  |
| ICD9 | 30453 | 30453 - HALLUCINOGEN DEP-REMISS | Table 1 | Substance use - hallucinogen |  |
| ICD9 | 3046 | 3046 - DRUG DEPENDENCE NEC | Table 1 | Substance use - hallucinogen |  |
| ICD9 | 3053 | 3053 - HALLUCINOGEN ABUSE | Table 1 | Substance use - hallucinogen |  |
| ICD9 | 30530 | 30530 - HALLUCINOGEN ABUSE-NOS | Table 1 | Substance use - hallucinogen |  |
| ICD9 | 30531 | 30531 - HALLUCINOGEN ABUSE-CONT | Table 1 | Substance use - hallucinogen |  |
| ICD9 | 30532 | 30532 - HALLUCINOGEN ABUSE-EPIS | Table 1 | Substance use - hallucinogen |  |
| ICD9 | 30533 | 30533 - HALLUCINOG ABUSE-REMISS | Table 1 | Substance use - hallucinogen |  |
| ICD10 | F16 | F16 - Hallucinogen related disorders | Table 1 | Substance use - hallucinogen |  |
| ICD10 | F161 | F161 - Hallucinogen abuse | Table 1 | Substance use - hallucinogen |  |
| ICD10 | F1610 | F1610 - Hallucinogen abuse, uncomplicated | Table 1 | Substance use - hallucinogen |  |
| ICD10 | F1611 | F1611 - Hallucinogen abuse, in remission | Table 1 | Substance use - hallucinogen |  |
| ICD10 | F1612 | F1612 - Hallucinogen abuse with intoxication | Table 1 | Substance use - hallucinogen |  |
| ICD10 | F16120 | F16120 - Hallucinogen abuse with intoxication, uncomplicated | Table 1 | Substance use - hallucinogen |  |
| ICD10 | F16121 | F16121 - Hallucinogen abuse with intoxication with delirium | Table 1 | Substance use - hallucinogen |  |
| ICD10 | F16122 | F16122 - Hallucinogen abuse w intoxication w perceptual disturbance | Table 1 | Substance use - hallucinogen |  |
| ICD10 | F16129 | F16129 - Hallucinogen abuse with intoxication, unspecified | Table 1 | Substance use - hallucinogen |  |
| ICD10 | F1614 | F1614 - Hallucinogen abuse with hallucinogen-induced mood disorder | Table 1 | Substance use - hallucinogen |  |
| ICD10 | F1615 | F1615 - Hallucinogen abuse w hallucinogen-induced psychotic disorder | Table 1 | Substance use - hallucinogen |  |
| ICD10 | F16150 | F16150 - Hallucinogen abuse w psychotic disorder w delusions | Table 1 | Substance use - hallucinogen |  |
| ICD10 | F16151 | F16151 - Hallucinogen abuse w psychotic disorder w hallucinations | Table 1 | Substance use - hallucinogen |  |
| ICD10 | F16159 | F16159 - Hallucinogen abuse w psychotic disorder, unsp | Table 1 | Substance use - hallucinogen |  |
| ICD10 | F1618 | F1618 - Hallucinogen abuse with other hallucinogen-induced disorder | Table 1 | Substance use - hallucinogen |  |
| ICD10 | F16180 | F16180 - Hallucinogen abuse w hallucinogen-induced anxiety disorder | Table 1 | Substance use - hallucinogen |  |
| ICD10 | F16183 | F16183 - Hallucign abuse w hallucign persisting perception disorder | Table 1 | Substance use - hallucinogen |  |
| ICD10 | F16188 | F16188 - Hallucinogen abuse with other hallucinogen-induced disorder | Table 1 | Substance use - hallucinogen |  |
| ICD10 | F1619 | F1619 - Hallucinogen abuse with unsp hallucinogen-induced disorder | Table 1 | Substance use - hallucinogen |  |
| ICD10 | F162 | F162 - Hallucinogen dependence | Table 1 | Substance use - hallucinogen |  |
| ICD10 | F1620 | F1620 - Hallucinogen dependence, uncomplicated | Table 1 | Substance use - hallucinogen |  |
| ICD10 | F1621 | F1621 - Hallucinogen dependence, in remission | Table 1 | Substance use - hallucinogen |  |
| ICD10 | F1622 | F1622 - Hallucinogen dependence with intoxication | Table 1 | Substance use - hallucinogen |  |
| ICD10 | F16220 | F16220 - Hallucinogen dependence with intoxication, uncomplicated | Table 1 | Substance use - hallucinogen |  |
| ICD10 | F16221 | F16221 - Hallucinogen dependence with intoxication with delirium | Table 1 | Substance use - hallucinogen |  |
| ICD10 | F16229 | F16229 - Hallucinogen dependence with intoxication, unspecified | Table 1 | Substance use - hallucinogen |  |
| ICD10 | F1624 | F1624 - Hallucinogen dependence w hallucinogen-induced mood disorder | Table 1 | Substance use - hallucinogen |  |
| ICD10 | F1625 | F1625 - Hallucinogen dependence w psychotic disorder | Table 1 | Substance use - hallucinogen |  |
| ICD10 | F16250 | F16250 - Hallucinogen dependence w psychotic disorder w delusions | Table 1 | Substance use - hallucinogen |  |
| ICD10 | F16251 | F16251 - Hallucinogen dependence w psychotic disorder w hallucin | Table 1 | Substance use - hallucinogen |  |
| ICD10 | F16259 | F16259 - Hallucinogen dependence w psychotic disorder, unsp | Table 1 | Substance use - hallucinogen |  |
| ICD10 | F1628 | F1628 - Hallucinogen dependence w oth hallucinogen-induced disorder | Table 1 | Substance use - hallucinogen |  |
| ICD10 | F16280 | F16280 - Hallucinogen dependence w anxiety disorder | Table 1 | Substance use - hallucinogen |  |
| ICD10 | F16283 | F16283 - Hallucign depend w hallucign persisting perception disorder | Table 1 | Substance use - hallucinogen |  |
| ICD10 | F16288 | F16288 - Hallucinogen dependence w oth hallucinogen-induced disorder | Table 1 | Substance use - hallucinogen |  |
| ICD10 | F1629 | F1629 - Hallucinogen dependence w unsp hallucinogen-induced disorder | Table 1 | Substance use - hallucinogen |  |
| ICD10 | F169 | F169 - Hallucinogen use, unspecified | Table 1 | Substance use - hallucinogen |  |
| ICD10 | F1690 | F1690 - Hallucinogen use, unspecified, uncomplicated | Table 1 | Substance use - hallucinogen |  |
| ICD10 | F1692 | F1692 - Hallucinogen use, unspecified with intoxication | Table 1 | Substance use - hallucinogen |  |
| ICD10 | F16920 | F16920 - Hallucinogen use, unsp with intoxication, uncomplicated | Table 1 | Substance use - hallucinogen |  |
| ICD10 | F16921 | F16921 - Hallucinogen use, unsp with intoxication with delirium | Table 1 | Substance use - hallucinogen |  |
| ICD10 | F16929 | F16929 - Hallucinogen use, unspecified with intoxication, unspecified | Table 1 | Substance use - hallucinogen |  |
| ICD10 | F1694 | F1694 - Hallucinogen use, unsp w hallucinogen-induced mood disorder | Table 1 | Substance use - hallucinogen |  |
| ICD10 | F1695 | F1695 - Hallucinogen use, unsp w psychotic disorder | Table 1 | Substance use - hallucinogen |  |
| ICD10 | F16950 | F16950 - Hallucinogen use, unsp w psychotic disorder w delusions | Table 1 | Substance use - hallucinogen |  |
| ICD10 | F16951 | F16951 - Hallucinogen use, unsp w psychotic disorder w hallucinations | Table 1 | Substance use - hallucinogen |  |
| ICD10 | F16959 | F16959 - Hallucinogen use, unsp w psychotic disorder, unsp | Table 1 | Substance use - hallucinogen |  |
| ICD10 | F1698 | F1698 - Hallucinogen use, unsp w oth hallucinogen-induced disorder | Table 1 | Substance use - hallucinogen |  |
| ICD10 | F16980 | F16980 - Hallucinogen use, unsp w anxiety disorder | Table 1 | Substance use - hallucinogen |  |
| ICD10 | F16983 | F16983 - Hallucign use, unsp w hallucign persist perception disorder | Table 1 | Substance use - hallucinogen |  |
| ICD10 | F16988 | F16988 - Hallucinogen use, unsp w oth hallucinogen-induced disorder | Table 1 | Substance use - hallucinogen |  |
| ICD10 | F1699 | F1699 - Hallucinogen use, unsp w unsp hallucinogen-induced disorder | Table 1 | Substance use - hallucinogen |  |
| ICD9 | 304 | 304 - DRUG DEPENDENCE | Table 1 | Substance use - nonspecific |  |
| ICD9 | 30460 | 30460 - DRUG DEP NEC-UNSPEC | Table 1 | Substance use - nonspecific |  |
| ICD9 | 30461 | 30461 - DRUG DEP NEC-CONT | Table 1 | Substance use - nonspecific |  |
| ICD9 | 30462 | 30462 - DRUG DEP NEC-EPISODIC | Table 1 | Substance use - nonspecific |  |
| ICD9 | 30463 | 30463 - DRUG DEP NEC-IN REMISS | Table 1 | Substance use - nonspecific |  |
| ICD9 | 3048 | 3048 - COMB DRUG DEPENDENCE NEC | Table 1 | Substance use - nonspecific |  |
| ICD9 | 30480 | 30480 - COMB DRUG DEP NEC-UNSPEC | Table 1 | Substance use - nonspecific |  |
| ICD9 | 30481 | 30481 - COMB DRUG DEP NEC-CONT | Table 1 | Substance use - nonspecific |  |
| ICD9 | 30482 | 30482 - COMB DRUG DEP NEC-EPIS | Table 1 | Substance use - nonspecific |  |
| ICD9 | 30483 | 30483 - COMB DRUG DEP NEC-REMISS | Table 1 | Substance use - nonspecific |  |
| ICD9 | 3049 | 3049 - DRUG DEPENDENCE NOS | Table 1 | Substance use - nonspecific |  |
| ICD9 | 30490 | 30490 - DRUG DEP NOS-UNSPEC | Table 1 | Substance use - nonspecific |  |
| ICD9 | 30491 | 30491 - DRUG DEP NOS-CONT | Table 1 | Substance use - nonspecific |  |
| ICD9 | 30492 | 30492 - DRUG DEP NOS-EPISODIC | Table 1 | Substance use - nonspecific |  |
| ICD9 | 30493 | 30493 - DRUG DEP NOS-REMISS | Table 1 | Substance use - nonspecific |  |
| ICD9 | 305 | 305 - NONDEPENDENT DRUG ABUSE | Table 1 | Substance use - nonspecific |  |
| ICD9 | 3059 | 3059 - DRUG ABUSE NEC & NOS | Table 1 | Substance use - nonspecific |  |
| ICD9 | 30590 | 30590 - DRUG ABUSE NEC-UNSPEC | Table 1 | Substance use - nonspecific |  |
| ICD9 | 30591 | 30591 - DRUG ABUSE NEC-CONT | Table 1 | Substance use - nonspecific |  |
| ICD9 | 30592 | 30592 - DRUG ABUSE NEC-EPIS | Table 1 | Substance use - nonspecific |  |
| ICD9 | 30593 | 30593 - DRUG ABUSE NEC-IN REMISS | Table 1 | Substance use - nonspecific |  |
| ICD9 | 3040 | 3040 - OPIOID TYPE DEPENDENCE | Table 1 | Substance use - opioid |  |
| ICD9 | 30400 | 30400 - OPIOID DEPENDENCE-UNSPEC | Table 1 | Substance use - opioid |  |
| ICD9 | 30401 | 30401 - OPIOID DEPENDENCE-CONT | Table 1 | Substance use - opioid |  |
| ICD9 | 30402 | 30402 - OPIOID DEPENDENCE-EPIS | Table 1 | Substance use - opioid |  |
| ICD9 | 30403 | 30403 - OPIOID DEPENDENCE-REMISS | Table 1 | Substance use - opioid |  |
| ICD9 | 3047 | 3047 - OPIOID/OTHER DRUG DEP | Table 1 | Substance use - opioid |  |
| ICD9 | 30470 | 30470 - OPIOID/OTHER DEP-UNSPEC | Table 1 | Substance use - opioid |  |
| ICD9 | 30471 | 30471 - OPIOID/OTHER DEP-CONT | Table 1 | Substance use - opioid |  |
| ICD9 | 30472 | 30472 - OPIOID/OTHER DEP-EPIS | Table 1 | Substance use - opioid |  |
| ICD9 | 30473 | 30473 - OPIOID/OTHER DEP-REMISS | Table 1 | Substance use - opioid |  |
| ICD9 | 3055 | 3055 - OPIOID ABUSE | Table 1 | Substance use - opioid |  |
| ICD9 | 30550 | 30550 - OPIOID ABUSE-UNSPEC | Table 1 | Substance use - opioid |  |
| ICD9 | 30551 | 30551 - OPIOID ABUSE-CONTINUOUS | Table 1 | Substance use - opioid |  |
| ICD9 | 30552 | 30552 - OPIOID ABUSE-EPISODIC | Table 1 | Substance use - opioid |  |
| ICD9 | 30553 | 30553 - OPIOID ABUSE-IN REMISS | Table 1 | Substance use - opioid |  |
| ICD10 | F11 | F11 - Opioid related disorders | Table 1 | Substance use - opioid |  |
| ICD10 | F111 | F111 - Opioid abuse | Table 1 | Substance use - opioid |  |
| ICD10 | F1110 | F1110 - Opioid abuse, uncomplicated | Table 1 | Substance use - opioid |  |
| ICD10 | F1111 | F1111 - Opioid abuse, in remission | Table 1 | Substance use - opioid |  |
| ICD10 | F1112 | F1112 - Opioid abuse with intoxication | Table 1 | Substance use - opioid |  |
| ICD10 | F11120 | F11120 - Opioid abuse with intoxication, uncomplicated | Table 1 | Substance use - opioid |  |
| ICD10 | F11121 | F11121 - Opioid abuse with intoxication delirium | Table 1 | Substance use - opioid |  |
| ICD10 | F11122 | F11122 - Opioid abuse with intoxication with perceptual disturbance | Table 1 | Substance use - opioid |  |
| ICD10 | F11129 | F11129 - Opioid abuse with intoxication, unspecified | Table 1 | Substance use - opioid |  |
| ICD10 | F1114 | F1114 - Opioid abuse with opioid-induced mood disorder | Table 1 | Substance use - opioid |  |
| ICD10 | F1115 | F1115 - Opioid abuse with opioid-induced psychotic disorder | Table 1 | Substance use - opioid |  |
| ICD10 | F11150 | F11150 - Opioid abuse w opioid-induced psychotic disorder w delusions | Table 1 | Substance use - opioid |  |
| ICD10 | F11151 | F11151 - Opioid abuse w opioid-induced psychotic disorder w hallucin | Table 1 | Substance use - opioid |  |
| ICD10 | F11159 | F11159 - Opioid abuse with opioid-induced psychotic disorder, unsp | Table 1 | Substance use - opioid |  |
| ICD10 | F1118 | F1118 - Opioid abuse with other opioid-induced disorder | Table 1 | Substance use - opioid |  |
| ICD10 | F11181 | F11181 - Opioid abuse with opioid-induced sexual dysfunction | Table 1 | Substance use - opioid |  |
| ICD10 | F11182 | F11182 - Opioid abuse with opioid-induced sleep disorder | Table 1 | Substance use - opioid |  |
| ICD10 | F11188 | F11188 - Opioid abuse with other opioid-induced disorder | Table 1 | Substance use - opioid |  |
| ICD10 | F1119 | F1119 - Opioid abuse with unspecified opioid-induced disorder | Table 1 | Substance use - opioid |  |
| ICD10 | F112 | F112 - Opioid dependence | Table 1 | Substance use - opioid |  |
| ICD10 | F1120 | F1120 - Opioid dependence, uncomplicated | Table 1 | Substance use - opioid |  |
| ICD10 | F1121 | F1121 - Opioid dependence, in remission | Table 1 | Substance use - opioid |  |
| ICD10 | F1122 | F1122 - Opioid dependence with intoxication | Table 1 | Substance use - opioid |  |
| ICD10 | F11220 | F11220 - Opioid dependence with intoxication, uncomplicated | Table 1 | Substance use - opioid |  |
| ICD10 | F11221 | F11221 - Opioid dependence with intoxication delirium | Table 1 | Substance use - opioid |  |
| ICD10 | F11222 | F11222 - Opioid dependence w intoxication with perceptual disturbance | Table 1 | Substance use - opioid |  |
| ICD10 | F11229 | F11229 - Opioid dependence with intoxication, unspecified | Table 1 | Substance use - opioid |  |
| ICD10 | F1123 | F1123 - Opioid dependence with withdrawal | Table 1 | Substance use - opioid |  |
| ICD10 | F1124 | F1124 - Opioid dependence with opioid-induced mood disorder | Table 1 | Substance use - opioid |  |
| ICD10 | F1125 | F1125 - Opioid dependence with opioid-induced psychotic disorder | Table 1 | Substance use - opioid |  |
| ICD10 | F11250 | F11250 - Opioid depend w opioid-induc psychotic disorder w delusions | Table 1 | Substance use - opioid |  |
| ICD10 | F11251 | F11251 - Opioid depend w opioid-induc psychotic disorder w hallucin | Table 1 | Substance use - opioid |  |
| ICD10 | F11259 | F11259 - Opioid dependence w opioid-induced psychotic disorder, unsp | Table 1 | Substance use - opioid |  |
| ICD10 | F1128 | F1128 - Opioid dependence with other opioid-induced disorder | Table 1 | Substance use - opioid |  |
| ICD10 | F11281 | F11281 - Opioid dependence with opioid-induced sexual dysfunction | Table 1 | Substance use - opioid |  |
| ICD10 | F11282 | F11282 - Opioid dependence with opioid-induced sleep disorder | Table 1 | Substance use - opioid |  |
| ICD10 | F11288 | F11288 - Opioid dependence with other opioid-induced disorder | Table 1 | Substance use - opioid |  |
| ICD10 | F1129 | F1129 - Opioid dependence with unspecified opioid-induced disorder | Table 1 | Substance use - opioid |  |
| ICD10 | F119 | F119 - Opioid use, unspecified | Table 1 | Substance use - opioid |  |
| ICD10 | F1190 | F1190 - Opioid use, unspecified, uncomplicated | Table 1 | Substance use - opioid |  |
| ICD10 | F1192 | F1192 - Opioid use, unspecified with intoxication | Table 1 | Substance use - opioid |  |
| ICD10 | F11920 | F11920 - Opioid use, unspecified with intoxication, uncomplicated | Table 1 | Substance use - opioid |  |
| ICD10 | F11921 | F11921 - Opioid use, unspecified with intoxication delirium | Table 1 | Substance use - opioid |  |
| ICD10 | F11922 | F11922 - Opioid use, unsp w intoxication with perceptual disturbance | Table 1 | Substance use - opioid |  |
| ICD10 | F11929 | F11929 - Opioid use, unspecified with intoxication, unspecified | Table 1 | Substance use - opioid |  |
| ICD10 | F1193 | F1193 - Opioid use, unspecified with withdrawal | Table 1 | Substance use - opioid |  |
| ICD10 | F1194 | F1194 - Opioid use, unspecified with opioid-induced mood disorder | Table 1 | Substance use - opioid |  |
| ICD10 | F1195 | F1195 - Opioid use, unsp with opioid-induced psychotic disorder | Table 1 | Substance use - opioid |  |
| ICD10 | F11950 | F11950 - Opioid use, unsp w opioid-induc psych disorder w delusions | Table 1 | Substance use - opioid |  |
| ICD10 | F11951 | F11951 - Opioid use, unsp w opioid-induc psych disorder w hallucin | Table 1 | Substance use - opioid |  |
| ICD10 | F11959 | F11959 - Opioid use, unsp w opioid-induced psychotic disorder, unsp | Table 1 | Substance use - opioid |  |
| ICD10 | F1198 | F1198 - Opioid use, unspecified with oth opioid-induced disorder | Table 1 | Substance use - opioid |  |
| ICD10 | F11981 | F11981 - Opioid use, unsp with opioid-induced sexual dysfunction | Table 1 | Substance use - opioid |  |
| ICD10 | F11982 | F11982 - Opioid use, unspecified with opioid-induced sleep disorder | Table 1 | Substance use - opioid |  |
| ICD10 | F11988 | F11988 - Opioid use, unspecified with other opioid-induced disorder | Table 1 | Substance use - opioid |  |
| ICD10 | F1199 | F1199 - Opioid use, unsp with unspecified opioid-induced disorder | Table 1 | Substance use - opioid |  |
| ICD10 | F12 | F12 - Cannabis related disorders | Table 1 | Substance use - opioid |  |
| ICD10 | F121 | F121 - Cannabis abuse | Table 1 | Substance use - opioid |  |
| ICD10 | F1210 | F1210 - Cannabis abuse, uncomplicated | Table 1 | Substance use - opioid |  |
| ICD10 | F1211 | F1211 - Cannabis abuse, in remission | Table 1 | Substance use - opioid |  |
| ICD10 | F1212 | F1212 - Cannabis abuse with intoxication | Table 1 | Substance use - opioid |  |
| ICD10 | F12120 | F12120 - Cannabis abuse with intoxication, uncomplicated | Table 1 | Substance use - opioid |  |
| ICD10 | F12121 | F12121 - Cannabis abuse with intoxication delirium | Table 1 | Substance use - opioid |  |
| ICD10 | F12122 | F12122 - Cannabis abuse with intoxication with perceptual disturbance | Table 1 | Substance use - opioid |  |
| ICD10 | F12129 | F12129 - Cannabis abuse with intoxication, unspecified | Table 1 | Substance use - opioid |  |
| ICD10 | F1215 | F1215 - Cannabis abuse with psychotic disorder | Table 1 | Substance use - opioid |  |
| ICD10 | F12150 | F12150 - Cannabis abuse with psychotic disorder with delusions | Table 1 | Substance use - opioid |  |
| ICD10 | F12151 | F12151 - Cannabis abuse with psychotic disorder with hallucinations | Table 1 | Substance use - opioid |  |
| ICD10 | F12159 | F12159 - Cannabis abuse with psychotic disorder, unspecified | Table 1 | Substance use - opioid |  |
| ICD10 | F1218 | F1218 - Cannabis abuse with other cannabis-induced disorder | Table 1 | Substance use - opioid |  |
| ICD10 | F12180 | F12180 - Cannabis abuse with cannabis-induced anxiety disorder | Table 1 | Substance use - opioid |  |
| ICD10 | F12188 | F12188 - Cannabis abuse with other cannabis-induced disorder | Table 1 | Substance use - opioid |  |
| ICD10 | F1219 | F1219 - Cannabis abuse with unspecified cannabis-induced disorder | Table 1 | Substance use - opioid |  |
| ICD10 | F122 | F122 - Cannabis dependence | Table 1 | Substance use - opioid |  |
| ICD10 | F1220 | F1220 - Cannabis dependence, uncomplicated | Table 1 | Substance use - opioid |  |
| ICD10 | F1221 | F1221 - Cannabis dependence, in remission | Table 1 | Substance use - opioid |  |
| ICD10 | F1222 | F1222 - Cannabis dependence with intoxication | Table 1 | Substance use - opioid |  |
| ICD10 | F12220 | F12220 - Cannabis dependence with intoxication, uncomplicated | Table 1 | Substance use - opioid |  |
| ICD10 | F12221 | F12221 - Cannabis dependence with intoxication delirium | Table 1 | Substance use - opioid |  |
| ICD10 | F12222 | F12222 - Cannabis dependence w intoxication w perceptual disturbance | Table 1 | Substance use - opioid |  |
| ICD10 | F12229 | F12229 - Cannabis dependence with intoxication, unspecified | Table 1 | Substance use - opioid |  |
| ICD10 | F1225 | F1225 - Cannabis dependence with psychotic disorder | Table 1 | Substance use - opioid |  |
| ICD10 | F12250 | F12250 - Cannabis dependence with psychotic disorder with delusions | Table 1 | Substance use - opioid |  |
| ICD10 | F12251 | F12251 - Cannabis dependence w psychotic disorder with hallucinations | Table 1 | Substance use - opioid |  |
| ICD10 | F12259 | F12259 - Cannabis dependence with psychotic disorder, unspecified | Table 1 | Substance use - opioid |  |
| ICD10 | F1228 | F1228 - Cannabis dependence with other cannabis-induced disorder | Table 1 | Substance use - opioid |  |
| ICD10 | F12280 | F12280 - Cannabis dependence with cannabis-induced anxiety disorder | Table 1 | Substance use - opioid |  |
| ICD10 | F12288 | F12288 - Cannabis dependence with other cannabis-induced disorder | Table 1 | Substance use - opioid |  |
| ICD10 | F1229 | F1229 - Cannabis dependence with unsp cannabis-induced disorder | Table 1 | Substance use - opioid |  |
| ICD10 | F129 | F129 - Cannabis use, unspecified | Table 1 | Substance use - opioid |  |
| ICD10 | F1290 | F1290 - Cannabis use, unspecified, uncomplicated | Table 1 | Substance use - opioid |  |
| ICD10 | F1292 | F1292 - Cannabis use, unspecified with intoxication | Table 1 | Substance use - opioid |  |
| ICD10 | F12920 | F12920 - Cannabis use, unspecified with intoxication, uncomplicated | Table 1 | Substance use - opioid |  |
| ICD10 | F12921 | F12921 - Cannabis use, unspecified with intoxication delirium | Table 1 | Substance use - opioid |  |
| ICD10 | F12922 | F12922 - Cannabis use, unsp w intoxication w perceptual disturbance | Table 1 | Substance use - opioid |  |
| ICD10 | F12929 | F12929 - Cannabis use, unspecified with intoxication, unspecified | Table 1 | Substance use - opioid |  |
| ICD10 | F1295 | F1295 - Cannabis use, unspecified with psychotic disorder | Table 1 | Substance use - opioid |  |
| ICD10 | F12950 | F12950 - Cannabis use, unsp with psychotic disorder with delusions | Table 1 | Substance use - opioid |  |
| ICD10 | F12951 | F12951 - Cannabis use, unsp w psychotic disorder with hallucinations | Table 1 | Substance use - opioid |  |
| ICD10 | F12959 | F12959 - Cannabis use, unsp with psychotic disorder, unspecified | Table 1 | Substance use - opioid |  |
| ICD10 | F1298 | F1298 - Cannabis use, unsp with other cannabis-induced disorder | Table 1 | Substance use - opioid |  |
| ICD10 | F12980 | F12980 - Cannabis use, unspecified with anxiety disorder | Table 1 | Substance use - opioid |  |
| ICD10 | F12988 | F12988 - Cannabis use, unsp with other cannabis-induced disorder | Table 1 | Substance use - opioid |  |
| ICD10 | F1299 | F1299 - Cannabis use, unsp with unsp cannabis-induced disorder | Table 1 | Substance use - opioid |  |
| ICD9 | 3044 | 3044 - AMPHETAMINE DEPENDENCE | Table 1 | Substance use - stimulant |  |
| ICD9 | 30440 | 30440 - AMPHETAMINE DEP-UNSPEC | Table 1 | Substance use - stimulant |  |
| ICD9 | 30441 | 30441 - AMPHETAMINE DEP-CONT | Table 1 | Substance use - stimulant |  |
| ICD9 | 30442 | 30442 - AMPHETAMINE DEP-EPIS | Table 1 | Substance use - stimulant |  |
| ICD9 | 30443 | 30443 - AMPHETAMINE DEP-REMISS | Table 1 | Substance use - stimulant |  |
| ICD9 | 3057 | 3057 - AMPHETAMINE ABUSE | Table 1 | Substance use - stimulant |  |
| ICD9 | 30570 | 30570 - AMPHETAMINE ABUSE-UNSPEC | Table 1 | Substance use - stimulant |  |
| ICD9 | 30571 | 30571 - AMPHETAMINE ABUSE-CONT | Table 1 | Substance use - stimulant |  |
| ICD9 | 30572 | 30572 - AMPHETAMINE ABUSE-EPIS | Table 1 | Substance use - stimulant |  |
| ICD9 | 30573 | 30573 - AMPHETAMINE ABUSE-REMISS | Table 1 | Substance use - stimulant |  |
| ICD10 | F15 | F15 - Other stimulant related disorders | Table 1 | Substance use - stimulant |  |
| ICD10 | F151 | F151 - Other stimulant abuse | Table 1 | Substance use - stimulant |  |
| ICD10 | F1510 | F1510 - Other stimulant abuse, uncomplicated | Table 1 | Substance use - stimulant |  |
| ICD10 | F1511 | F1511 - Other stimulant abuse, in remission | Table 1 | Substance use - stimulant |  |
| ICD10 | F1512 | F1512 - Other stimulant abuse with intoxication | Table 1 | Substance use - stimulant |  |
| ICD10 | F15120 | F15120 - Other stimulant abuse with intoxication, uncomplicated | Table 1 | Substance use - stimulant |  |
| ICD10 | F15121 | F15121 - Other stimulant abuse with intoxication delirium | Table 1 | Substance use - stimulant |  |
| ICD10 | F15122 | F15122 - Oth stimulant abuse w intoxication w perceptual disturbance | Table 1 | Substance use - stimulant |  |
| ICD10 | F15129 | F15129 - Other stimulant abuse with intoxication, unspecified | Table 1 | Substance use - stimulant |  |
| ICD10 | F1514 | F1514 - Other stimulant abuse with stimulant-induced mood disorder | Table 1 | Substance use - stimulant |  |
| ICD10 | F1515 | F1515 - Oth stimulant abuse w stimulant-induced psychotic disorder | Table 1 | Substance use - stimulant |  |
| ICD10 | F15150 | F15150 - Oth stimulant abuse w stim-induce psych disorder w delusions | Table 1 | Substance use - stimulant |  |
| ICD10 | F15151 | F15151 - Oth stimulant abuse w stim-induce psych disorder w hallucin | Table 1 | Substance use - stimulant |  |
| ICD10 | F15159 | F15159 - Oth stimulant abuse w stim-induce psychotic disorder, unsp | Table 1 | Substance use - stimulant |  |
| ICD10 | F1518 | F1518 - Other stimulant abuse with other stimulant-induced disorder | Table 1 | Substance use - stimulant |  |
| ICD10 | F15180 | F15180 - Oth stimulant abuse with stimulant-induced anxiety disorder | Table 1 | Substance use - stimulant |  |
| ICD10 | F15181 | F15181 - Oth stimulant abuse w stimulant-induced sexual dysfunction | Table 1 | Substance use - stimulant |  |
| ICD10 | F15182 | F15182 - Other stimulant abuse with stimulant-induced sleep disorder | Table 1 | Substance use - stimulant |  |
| ICD10 | F15188 | F15188 - Other stimulant abuse with other stimulant-induced disorder | Table 1 | Substance use - stimulant |  |
| ICD10 | F1519 | F1519 - Other stimulant abuse with unsp stimulant-induced disorder | Table 1 | Substance use - stimulant |  |
| ICD10 | F152 | F152 - Other stimulant dependence | Table 1 | Substance use - stimulant |  |
| ICD10 | F1520 | F1520 - Other stimulant dependence, uncomplicated | Table 1 | Substance use - stimulant |  |
| ICD10 | F1521 | F1521 - Other stimulant dependence, in remission | Table 1 | Substance use - stimulant |  |
| ICD10 | F1522 | F1522 - Other stimulant dependence with intoxication | Table 1 | Substance use - stimulant |  |
| ICD10 | F15220 | F15220 - Other stimulant dependence with intoxication, uncomplicated | Table 1 | Substance use - stimulant |  |
| ICD10 | F15221 | F15221 - Other stimulant dependence with intoxication delirium | Table 1 | Substance use - stimulant |  |
| ICD10 | F15222 | F15222 - Oth stimulant dependence w intox w perceptual disturbance | Table 1 | Substance use - stimulant |  |
| ICD10 | F15229 | F15229 - Other stimulant dependence with intoxication, unspecified | Table 1 | Substance use - stimulant |  |
| ICD10 | F1523 | F1523 - Other stimulant dependence with withdrawal | Table 1 | Substance use - stimulant |  |
| ICD10 | F1524 | F1524 - Oth stimulant dependence w stimulant-induced mood disorder | Table 1 | Substance use - stimulant |  |
| ICD10 | F1525 | F1525 - Oth stimulant dependence w stim-induce psychotic disorder | Table 1 | Substance use - stimulant |  |
| ICD10 | F15250 | F15250 - Oth stim depend w stim-induce psych disorder w delusions | Table 1 | Substance use - stimulant |  |
| ICD10 | F15251 | F15251 - Oth stimulant depend w stim-induce psych disorder w hallucin | Table 1 | Substance use - stimulant |  |
| ICD10 | F15259 | F15259 - Oth stimulant depend w stim-induce psychotic disorder, unsp | Table 1 | Substance use - stimulant |  |
| ICD10 | F1528 | F1528 - Oth stimulant dependence with oth stimulant-induced disorder | Table 1 | Substance use - stimulant |  |
| ICD10 | F15280 | F15280 - Oth stimulant dependence w stim-induce anxiety disorder | Table 1 | Substance use - stimulant |  |
| ICD10 | F15281 | F15281 - Oth stimulant dependence w stim-induce sexual dysfunction | Table 1 | Substance use - stimulant |  |
| ICD10 | F15282 | F15282 - Oth stimulant dependence w stimulant-induced sleep disorder | Table 1 | Substance use - stimulant |  |
| ICD10 | F15288 | F15288 - Oth stimulant dependence with oth stimulant-induced disorder | Table 1 | Substance use - stimulant |  |
| ICD10 | F1529 | F1529 - Oth stimulant dependence w unsp stimulant-induced disorder | Table 1 | Substance use - stimulant |  |
| ICD10 | F159 | F159 - Other stimulant use, unspecified | Table 1 | Substance use - stimulant |  |
| ICD10 | F1590 | F1590 - Other stimulant use, unspecified, uncomplicated | Table 1 | Substance use - stimulant |  |
| ICD10 | F1592 | F1592 - Other stimulant use, unspecified with intoxication | Table 1 | Substance use - stimulant |  |
| ICD10 | F15920 | F15920 - Other stimulant use, unsp with intoxication, uncomplicated | Table 1 | Substance use - stimulant |  |
| ICD10 | F15921 | F15921 - Other stimulant use, unspecified with intoxication delirium | Table 1 | Substance use - stimulant |  |
| ICD10 | F15922 | F15922 - Oth stimulant use, unsp w intox w perceptual disturbance | Table 1 | Substance use - stimulant |  |
| ICD10 | F15929 | F15929 - Other stimulant use, unsp with intoxication, unspecified | Table 1 | Substance use - stimulant |  |
| ICD10 | F1593 | F1593 - Other stimulant use, unspecified with withdrawal | Table 1 | Substance use - stimulant |  |
| ICD10 | F1594 | F1594 - Oth stimulant use, unsp with stimulant-induced mood disorder | Table 1 | Substance use - stimulant |  |
| ICD10 | F1595 | F1595 - Oth stimulant use, unsp w stim-induce psychotic disorder | Table 1 | Substance use - stimulant |  |
| ICD10 | F15950 | F15950 - Oth stim use, unsp w stim-induce psych disorder w delusions | Table 1 | Substance use - stimulant |  |
| ICD10 | F15951 | F15951 - Oth stim use, unsp w stim-induce psych disorder w hallucin | Table 1 | Substance use - stimulant |  |
| ICD10 | F15959 | F15959 - Oth stimulant use, unsp w stim-induce psych disorder, unsp | Table 1 | Substance use - stimulant |  |
| ICD10 | F1598 | F1598 - Oth stimulant use, unsp with oth stimulant-induced disorder | Table 1 | Substance use - stimulant |  |
| ICD10 | F15980 | F15980 - Oth stimulant use, unsp w stimulant-induced anxiety disorder | Table 1 | Substance use - stimulant |  |
| ICD10 | F15981 | F15981 - Oth stimulant use, unsp w stim-induce sexual dysfunction | Table 1 | Substance use - stimulant |  |
| ICD10 | F15982 | F15982 - Oth stimulant use, unsp w stimulant-induced sleep disorder | Table 1 | Substance use - stimulant |  |
| ICD10 | F15988 | F15988 - Oth stimulant use, unsp with oth stimulant-induced disorder | Table 1 | Substance use - stimulant |  |
| ICD10 | F1599 | F1599 - Oth stimulant use, unsp with unsp stimulant-induced disorder | Table 1 | Substance use - stimulant |  |
| ICD9 | 3051 | Tobacco use disorder | Table 1 | Substance use - tobacco |  |
| ICD10 | Z720 | Smoking | Table 1 | Substance use - tobacco |  |
| ICD10 | F17200 | Nicotine dependence, unspecified, uncomplicated | Table 1 | Substance use - tobacco |  |
| ICD9 | 3051 | 3051 - TOBACCO USE DISORDER | Table 1 | Substance use - tobacco |  |
| ICD9 | 30510 | 30510 - tobacco abuse-unspec | Table 1 | Substance use - tobacco |  |
| ICD9 | 30511 | 30511 - tobacco abuse-continuou | Table 1 | Substance use - tobacco |  |
| ICD9 | 30512 | 30512 - tobacco abuse-episodic | Table 1 | Substance use - tobacco |  |
| ICD9 | 30513 | 30513 - tobacco abuse-in remiss | Table 1 | Substance use - tobacco |  |
| VizientCode | 819049 | Tracheostomy tube | Table 3 | Trache-ostomy |  |
| ICD9 | 3229 | 3229 - MENINGITIS NOS | Table 3 | Ventri-culitis/ Meningitis |  |
| ICD10 | G039 | G039 - Meningitis, unspecified | Table 3 | Ventri-culitis/ Meningitis |  |
| ICD9 | 3200 | 3200 - HEMOPHILUS MENINGITIS | Table 3 | Ventri-culitis/ Meningitis |  |
| ICD9 | 3201 | 3201 - PNEUMOCOCCAL MENINGITIS | Table 3 | Ventri-culitis/ Meningitis |  |
| ICD9 | 3202 | 3202 - STREPTOCOCCAL MENINGITIS | Table 3 | Ventri-culitis/ Meningitis |  |
| ICD9 | 3203 | 3203 - STAPH MENINGITIS | Table 3 | Ventri-culitis/ Meningitis |  |
| ICD9 | 3207 | 3207 - MENING IN BACT DCE NEC | Table 3 | Ventri-culitis/ Meningitis |  |
| ICD9 | 32082 | 32082 - GRAM NEG MENINGITIS NEC | Table 3 | Ventri-culitis/ Meningitis |  |
| ICD9 | 32089 | 32089 - OTH BACTERIAL MENINGITIS | Table 3 | Ventri-culitis/ Meningitis |  |
| ICD9 | 3209 | 3209 - BACTERIAL MENINGITIS NOS | Table 3 | Ventri-culitis/ Meningitis |  |
| ICD9 | 3211 | 3211 - MENING IN OTH FUNGAL DIS | Table 3 | Ventri-culitis/ Meningitis |  |
| ICD9 | 3212 | 3212 - MENING IN OTH VIRAL DIS | Table 3 | Ventri-culitis/ Meningitis |  |
| ICD9 | 3218 | 3218 - MENING IN NONBAC ORG NEC | Table 3 | Ventri-culitis/ Meningitis |  |
| ICD9 | 3220 | 3220 - NONPYOGENIC MENINGITIS | Table 3 | Ventri-culitis/ Meningitis |  |
| ICD9 | 3221 | 3221 - EOSINOPHILIC MENINGITIS | Table 3 | Ventri-culitis/ Meningitis |  |
| ICD9 | 3222 | 3222 - CHRONIC MENINGITIS | Table 3 | Ventri-culitis/ Meningitis |  |
| ICD10 | G000 | G000 - Hemophilus meningitis | Table 3 | Ventri-culitis/ Meningitis |  |
| ICD10 | G001 | G001 - Pneumococcal meningitis | Table 3 | Ventri-culitis/ Meningitis |  |
| ICD10 | G002 | G002 - Streptococcal meningitis | Table 3 | Ventri-culitis/ Meningitis |  |
| ICD10 | G003 | G003 - Staphylococcal meningitis | Table 3 | Ventri-culitis/ Meningitis |  |
| ICD10 | G008 | G008 - Other bacterial meningitis | Table 3 | Ventri-culitis/ Meningitis |  |
| ICD10 | G009 | G009 - Bacterial meningitis, unspecified | Table 3 | Ventri-culitis/ Meningitis |  |
| ICD10 | G01 | G01 - Meningitis in bacterial diseases classified elsewhere | Table 3 | Ventri-culitis/ Meningitis |  |
| ICD10 | G02 | G02 - Meningitis in oth infec/parastc diseases classd elswhr | Table 3 | Ventri-culitis/ Meningitis |  |
| ICD10 | G030 | G030 - Nonpyogenic meningitis | Table 3 | Ventri-culitis/ Meningitis |  |
| ICD10 | G031 | G031 - Chronic meningitis | Table 3 | Ventri-culitis/ Meningitis |  |
| ICD10 | G032 | G032 - Benign recurrent meningitis [Mollaret] | Table 3 | Ventri-culitis/ Meningitis |  |
| ICD10 | G038 | G038 - Meningitis due to other specified causes | Table 3 | Ventri-culitis/ Meningitis |  |
| ICD9 | 27661 | 27661 - TACO | Table 3 | Volume overload |  |
| ICD9 | 27669 | 27669 - FLUID OVERLOAD NEC | Table 3 | Volume overload |  |
| ICD10 | E8770 | E8770 - Fluid overload, unspecified | Table 3 | Volume overload |  |
| ICD10 | E8771 | E8771 - Transfusion associated circulatory overload | Table 3 | Volume overload |  |
| ICD10 | E8779 | E8779 - Other fluid overload | Table 3 | Volume overload |  |
| ICD9 | V452 | V452 - VENTRICULAR SHUNT STATUS | Table 3 | VP Shunt |  |
| ICD10 | Z982 | Z982 - Presence of cerebrospinal fluid drainage device | Table 3 | VP Shunt |  |
| VizientCode |  | Transcranial Dopplers | Table 3 |  |  |
| ICD9 | 00845 | 00845 - C. DIFFICILE ENTERITIS | Table 3 | C. Diff |  |
| ICD10 | A047 | A047 - Enterocolitis due to Clostridium difficile | Table 3 | C. Diff |  |
| ICD10 | A0471 | A0471 - Enterocolitis due to Clostridium difficile, recurrent | Table 3 | C. Diff |  |
| ICD10 | A0472 | A0472 - Enterocolitis d/t Clostridium difficile, not spcf as recur | Table 3 | C. Diff |  |
| ICD9 | 410 | 410 - AMI | Table 3 | AMI |  |
| ICD9 | 4100 | 4100 - AMI ANTEROLATERAL WALL | Table 3 | AMI |  |
| ICD9 | 41000 | 41000 - ANTEROLAT AMI-EPIS NOS | Table 3 | AMI |  |
| ICD9 | 41001 | 41001 - ANTEROLAT AMI-INITIAL | Table 3 | AMI |  |
| ICD9 | 41002 | 41002 - ANTEROLATERAL AMI-SUBSQ | Table 3 | AMI |  |
| ICD9 | 4101 | 4101 - AMI ANTERIOR WALL NEC | Table 3 | AMI |  |
| ICD9 | 41010 | 41010 - ANT AMI NEC-EPISODE NOS | Table 3 | AMI |  |
| ICD9 | 41011 | 41011 - ANT AMI NEC-INITIAL | Table 3 | AMI |  |
| ICD9 | 41012 | 41012 - ANT AMI NEC-SUBSEQUENT | Table 3 | AMI |  |
| ICD9 | 4102 | 4102 - AMI INFEROLATERAL WALL | Table 3 | AMI |  |
| ICD9 | 41020 | 41020 - INFEROLAT AMI-EPIS NOS | Table 3 | AMI |  |
| ICD9 | 41021 | 41021 - INFEROLAT AMI-INITIAL | Table 3 | AMI |  |
| ICD9 | 41022 | 41022 - INFEROLAT AMI-SUBSQ | Table 3 | AMI |  |
| ICD9 | 4103 | 4103 - AMI INFEROPOSTERIOR WALL | Table 3 | AMI |  |
| ICD9 | 41030 | 41030 - INFEROPOST AMI-EPIS NOS | Table 3 | AMI |  |
| ICD9 | 41031 | 41031 - INFEROPOST AMI-INITIAL | Table 3 | AMI |  |
| ICD9 | 41032 | 41032 - INFEROPOST AMI-SUBSQ | Table 3 | AMI |  |
| ICD9 | 4104 | 4104 - AMI INFERIOR WALL NEC | Table 3 | AMI |  |
| ICD9 | 41040 | 41040 - INF AMI NEC-EPISODE NOS | Table 3 | AMI |  |
| ICD9 | 41041 | 41041 - INF AMI NEC-INITIAL | Table 3 | AMI |  |
| ICD9 | 41042 | 41042 - INF AMI NEC-SUBSEQUENT | Table 3 | AMI |  |
| ICD9 | 4105 | 4105 - AMI LATERAL WALL NEC | Table 3 | AMI |  |
| ICD9 | 41050 | 41050 - LAT AMI NEC-EPISODE NOS | Table 3 | AMI |  |
| ICD9 | 41051 | 41051 - LAT AMI NEC-INITIAL | Table 3 | AMI |  |
| ICD9 | 41052 | 41052 - LAT AMI NEC-SUBSEQUENT | Table 3 | AMI |  |
| ICD9 | 4106 | 4106 - TRUE POSTERIOR INFARCT | Table 3 | AMI |  |
| ICD9 | 41060 | 41060 - POST AMI-EPISODE NOS | Table 3 | AMI |  |
| ICD9 | 41061 | 41061 - POSTERIOR AMI-INITIAL | Table 3 | AMI |  |
| ICD9 | 41062 | 41062 - POSTERIOR AMI-SUBSQ | Table 3 | AMI |  |
| ICD9 | 4107 | 4107 - SUBENDOCARDIAL INFARCT | Table 3 | AMI |  |
| ICD9 | 41070 | 41070 - SUBEND INFARCT-EPIS NOS | Table 3 | AMI |  |
| ICD9 | 41071 | 41071 - SUBEND INFARCT-INITIAL | Table 3 | AMI |  |
| ICD9 | 41072 | 41072 - SUBEND INFARCT-SUBSQ | Table 3 | AMI |  |
| ICD9 | 4108 | 4108 - MYOCARDIAL INFARCT NEC | Table 3 | AMI |  |
| ICD9 | 41080 | 41080 - AMI NEC-EPISODE NOS | Table 3 | AMI |  |
| ICD9 | 41081 | 41081 - AMI NEC-INITIAL EPISODE | Table 3 | AMI |  |
| ICD9 | 41082 | 41082 - AMI NEC-SUBSEQUENT | Table 3 | AMI |  |
| ICD9 | 4109 | 4109 - MYOCARDIAL INFARCT NOS | Table 3 | AMI |  |
| ICD9 | 41090 | 41090 - AMI NOS-EPISODE NOS | Table 3 | AMI |  |
| ICD9 | 41091 | 41091 - AMI NOS-INITIAL EPISODE | Table 3 | AMI |  |
| ICD9 | 41092 | 41092 - AMI NOS-SUBSEQUENT | Table 3 | AMI |  |
| ICD10 | I21 | I21 - STEMI & NSTEMI mocard infrc | Table 3 | AMI |  |
| ICD10 | I210 | I210 - ST elevation (STEMI) myocardial infarction of anterior wall | Table 3 | AMI |  |
| ICD10 | I2101 | I2101 - STEMI involving left main coronary artery | Table 3 | AMI |  |
| ICD10 | I2102 | I2102 - STEMI involving left anterior descending coronary artery | Table 3 | AMI |  |
| ICD10 | I2109 | I2109 - STEMI involving oth coronary artery of anterior wall | Table 3 | AMI |  |
| ICD10 | I211 | I211 - ST elevation (STEMI) myocardial infarction of inferior wall | Table 3 | AMI |  |
| ICD10 | I2111 | I2111 - STEMI involving right coronary artery | Table 3 | AMI |  |
| ICD10 | I2119 | I2119 - STEMI involving oth coronary artery of inferior wall | Table 3 | AMI |  |
| ICD10 | I212 | I212 - ST elevation (STEMI) myocardial infarction of other sites | Table 3 | AMI |  |
| ICD10 | I2121 | I2121 - STEMI involving left circumflex coronary artery | Table 3 | AMI |  |
| ICD10 | I2129 | I2129 - STEMI involving oth sites | Table 3 | AMI |  |
| ICD10 | I213 | I213 - ST elevation (STEMI) myocardial infarction of unsp site | Table 3 | AMI |  |
| ICD10 | I214 | I214 - Non-ST elevation (NSTEMI) myocardial infarction | Table 3 | AMI |  |
| ICD10 | I219 | I219 - Acute myocardial infarction, unspecified | Table 3 | AMI |  |
| ICD10 | I21A1 | I21A1 - Myocardial infarction type 2 | Table 3 | AMI |  |
| ICD10 | I21A9 | I21A9 - Other myocardial infarction type | Table 3 | AMI |  |
| ICD9 | V461 | V461 - DEPENDENCE ON RESPIRATOR | Table 3 | Ventilator |  |
| ICD9 | V4611 | V4611 - RESPIRATOR DEPEND STATUS | Table 3 | Ventilator |  |
| ICD9 | V4613 | V4613 - WEANING FROM RESPIRATOR | Table 3 | Ventilator |  |
| ICD9 | V4614 | V4614 - MECH COMP RESPIRATOR | Table 3 | Ventilator |  |
| ICD10 | Z991 | Z991 - Dependence on respirator | Table 3 | Ventilator |  |
| ICD10 | Z9911 | Z9911 - Dependence on respirator [ventilator] status | Table 3 | Ventilator |  |
